# Synthesis and performance of binder-free porous carbon electrodes in electrochemical capacitors†

**DOI:** 10.1039/d3ta04971j

**Published:** 2024-02-14

**Authors:** Anetta Platek-Mielczarek, Adrian Beda, Krzysztof Fic, Camelia Matei Ghimbeu

**Affiliations:** a Poznan University of Technology, Institute of Chemistry and Technical Electrochemistry Berdychowo 4 60-965 Poznan Poland krzysztof.fic@put.poznan.pl; b Laboratory for Multiphase Thermofluidics and Surface Nanoengineering, Department of Mechanical and Process Engineering, ETH Zurich Sonneggstrasse 3 Zurich Switzerland; c Université de Haute-Alsace, Institut de Science des Matériaux de Mulhouse (IS2M), CNRS, UMR 7361 Mulhouse F-68100 France camelia.ghimbeu@uha.fr; d Université de Strasbourg Strasbourg F-67081 France; e Réseau sur le Stockage Electrochimique de l'Energie (RS2E), CNRS FR3459 33 Rue Saint Leu Amiens Cedex 80039 France

## Abstract

Porous binder-free carbon electrodes were obtained by impregnating cellulose filter papers with phenolic resin through soft-salt template synthesis and thermal pyrolysis. These self-standing electrodes were used directly in a supercapacitor device. To understand the impacts of filter paper (FP) thickness on carbon filter paper (CFP) morphology, porosity, and surface functionalities, five different materials were examined. The CFP electrode thickness was adjusted linearly with the FP thickness to produce electrodes ranging from 100 to ∼800 μm. As the thickness of the CFP increased, there was an increase in the specific surface area and oxygen-based functionalities. Electrochemical testing in a 1 M KOH aqueous electrolyte demonstrated that electrode thickness played a key role in electrochemical capacitor (EC) performance, *i.e.*, capacitance enhances linearly when the electrode becomes thinner. For low thicknesses (<280 μm), the capacitance and rate capability decreased slightly with increasing thickness; for high thicknesses, the performance drastically degraded despite the high specific surface area and oxygen surface functionalities. This effect was amplified at high regimes, indicating that high electrode thicknesses and fiber diameters limited electrolyte diffusion in the applied synthesis conditions. Thus, the high material porosity was inaccessible to ion adsorption. Consequently, thinner electrodes showed the highest capacitance (197 F g^−1^ at 0.1 A g^−1^) and rate capability (81%) values, exceeding those of their traditional binder-electrode counterparts prepared under similar conditions. Finally, for alkali metal hydroxide solutions, 1 M KOH exhibited a cation match with the salt template (KCl), while CsOH achieved additional capacitance retention (88% at 10 A g^−1^). Complex ion adsorption mechanisms were observed through a quartz crystal microbalance.

## Abstract 2

Porous binder-free carbon electrodes were obtained by using cellulose filter paper through soft-salt template synthesis. The electrode thickness could be tuned, and a linear relationship with the specific capacitance in supercapacitors was established.

## Graphical abstract

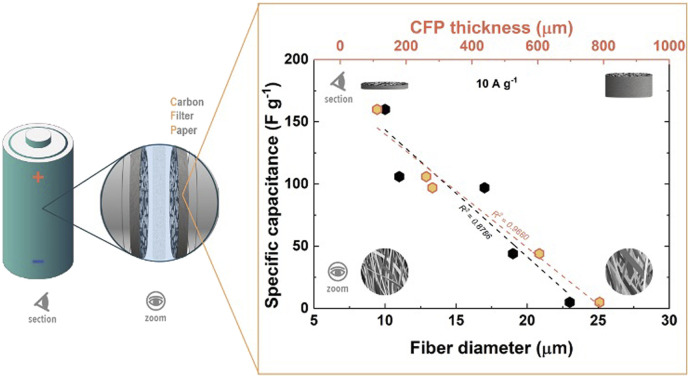

## 1. Introduction

Electrochemical capacitors (ECs) are energy storage devices with high power densities and long cycle life.^1^ ECs can deliver energy in a short time, making them ideal for applications requiring high power output, such as electric vehicles, renewable energy systems (power peak smoothing) and portable electronics. The performance attributes of ECs are mainly determined by their electrode materials, which store energy through the physical adsorption of electrolyte ions at the electrode/electrolyte interface.^2^ The ideal electrode material for electrochemical capacitors should have a developed specific surface area, high electronic conductivity, good chemical stability, and low ion diffusion resistance.^3^ Activated carbon (AC), as a material that fulfills these criteria, is abundant and inexpensive.^4^ ACs can be produced from a wide range of precursors, such as coal, coconut shell, and biomass, allowing their textural, structural, and chemical properties to be tailored.^5^ The high specific surface areas of ACs provide many accessible active sites for electric double-layer formation, whereas their porosity allows for efficient electrode bulk penetration by electrolyte.^6^ However, traditional methods for fabricating AC electrodes involve the use of polymer binders to form self-standing electrode composite films.^7^ Binders limit the accessibility of active sites for ion adsorption and decrease the overall capacitance and rate handling ability of the device. Moreover, binders can be susceptible to extreme temperature conditions, limiting the mechanical integrity of the electrode and its chemical resistance over time.^8^

With a recent trend for sustainable research and development, fluorine-containing binders are being replaced by environmentally friendly binders.^9^ Using a binder is necessary for holding carbon particles together and attaching them to a current collector.^10^ However, avoiding the binder improves the electrode accessibility by the electrolyte and the performance. To address this issue, researchers have explored the application of binder-free electrodes. Carbon cloth, felt, or paper provide multiple textural characteristics for flexible electrodes.^11,12^ These materials allow for the study of long-term EC behavior excluding binder decomposition.^13^ Moreover, they can be successfully employed as active material substrates combined with conducting polymers.^14^ However, advanced material science researchers are seeking easy and facile electrode fabrication processes using inexpensive and/or waste precursors.^15^

Binder-free electrodes can be fabricated using various techniques, including direct assembly, freeze-drying, and template synthesis. Another approach for producing binder-free electrodes involves the active material pressing onto current collectors, usually in the form of mesh or foam.^16,17^ Direct synthesis of electrode material on metal^18^ or self-assembly process are employed as well.^19,20^ Freeze-drying is another technique that can be used to fabricate binder-free electrodes. In this method, an activated carbon slurry is frozen and dried under vacuum conditions, resulting in a porous 3D structure.^21^ An alternative method is to use template synthesis, where a sacrificial template material is used to create a porous carbon structure. Most of this synthesis requires template removal to obtain a porous binder-free carbon electrode. Vapor phase polymerization with a porous template (CaCO_3_) forms binder-free electrodes made of a conductive polymer (polypyrrole, PPy) instead of carbon.^22^ This approach requires several synthesis steps, such as CaCO_3_ particle synthesis and preparation, PPy synthesis, and solid-state template removal. These electrodes have 3D structures, allowing for better ion diffusion in the electrode bulk. Conversely, the templating method does not require the use of a rigid template skeleton; to date, it employs a polymer and a sacrificial soft template that turns into carbon with developed porosity by thermal pyrolysis.^23^ This approach enables to avoid the template dissolution step *via* chemical etching. Moreover, the salt-template approach has emerged as an efficient synthesis method for finely tuning the carbon pore size and surface area, enabling high capacitance, retention and cycle life.^24^ The removal and recovery of the template by simple water washing and its reuse has attracted additional interest.^25,26^

Recently, advanced approaches for obtaining binder-free electrodes for electrochemical capacitors have been explored. Interestingly, filter paper can be used as a skeleton for binder-free electrodes made of single and multiwalled carbon nanotubes.^27^ These electrodes are prepared through the layer-by-layer filtration of the active material (carbon nanotubes) over filter paper doped with polyaniline (PANI). These electrodes exhibit good electrochemical performance when combined with a quasi solid electrolyte (H_2_SO_4_–polyvinyl alcohol (PVA) gel), and they are mechanically stable. Due to their intertwined structures, carbon nanotubes can form binder-free electrodes by filtration without mechanical support.^28^ For such mesoporous carbon, the absence of a binder significantly increases the specific surface area of the electrode, exhibiting values on the level of 100 m^2^ g^−1^. If dispersed on a membrane, one method is to separate the electrode material by the dissolution of filter paper (usually cellulose-based).^29^ This treatment results in a binder-free carbon nanotube electrode.

In summary, binder-free activated carbon electrodes have shown promising performance. They can offer higher specific capacitance, better rate performance, and longer cycle life than traditional binder-based electrodes. When the fabrication process is sufficiently easy, they can conserve the time and resources needed for EC assembly.

Therefore, in this work, we focus on a one-pot synthesis that combines filter papers and impregnation with phenolic resin and soft-salt templates to obtain porous carbon electrodes with tailored thicknesses and microtextures and excellent electrochemical properties. An optimized synthesis protocol is described, leading to self-standing carbon electrodes with foreseeable properties. The formation of thin (∼100 μm) to very thick electrodes (∼800 μm) is demonstrated, and the impacts of electrode thicknesses, porosities, and functionalities are systematically investigated. Since synthesized carbon electrodes do not contain a binder, several relationships between material properties and electrochemical performance have been established. Insights are provided into electrolyte ion adsorption mechanisms on carbon filter paper using microscale sensitive device, *i.e.*, electrochemical quartz crystal microbalance (EQCM). Furthermore, carbon binder-free electrodes exhibit very good cycling performance, with 10 000 cycles with 90% capacitance retention and maintained mechanical integrity after the electrochemical test.

## 2. Experimental procedure

### 2.1. Materials synthesis

**Fig. 1:**
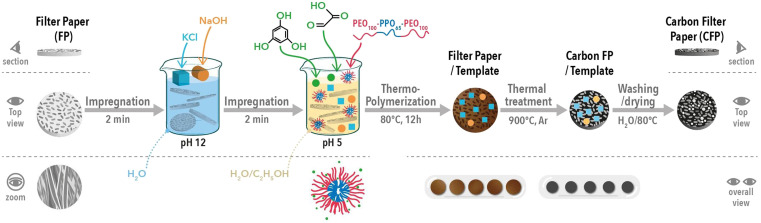
Synthesis of CFP from cellulose FP using a soft-salt templating method.

Binder-free electrodes were prepared according to the synthesis procedure presented in Fig. 1. The cellulose filter papers (FP) were purchased from Rotilabo-Rundfilter (FP_195) and Whatman (FP_390, FP_430, FP_750 and FP_1000). Thus, five different FPs were selected; the number present in the description of the sample's name denotes the initial thickness of the precursor. All their characteristics are presented in Table S1 and Fig. S1 in the ESI.† The general synthesis technique is based on impregnating the filter paper with solutions developed previously to obtain porous carbon materials by the soft-salt template approach.^30,31^ The use of a soft-template allowed mesopore generation, while salt induced micropore formation.

In the first step, the FPs cut at the desired diameter (14 mm) were first impregnated for two minutes in an aqueous solution of pH 12 that contained a mixture of KCl (1.118 g KCl + 5 mL H_2_O) and NaOH (0.250 g NaOH + 5 mL H_2_O).

Second, a phenolic resin solution was prepared; the solution was composed of phloroglucinol (0.415 g), glyoxylic acid (0.39 g) and Pluronic F127 soft template (0.8 g) and dispersed in 5 mL of Et–OH and 10 mL of H_2_O.^32^ The mixture was stirred for ∼30 min, and then the first impregnation solution (containing the salt) was added and stirred for a few more minutes (pH dropped to 5). The FPs were immersed for two minutes in the phenolic resin/salt solution to promote material porosity formation and improve the mechanical strength.^33^ The as-obtained impregnated FPs were dried overnight at 80 °C to induce resin polymerization and a high crosslinking degree. Furthermore, the impregnated films were heat treated at 900 °C (2 °C min^−1^) for 1 h in an inert atmosphere (15 L h^−1^ Ar) to thermally decompose the phenolic resin and soft-template with the formation of porous carbon.

The recovered carbon filter papers (CFPs) were subsequently washed with distilled hot water (80 °C) to remove the salt-template until a neutral pH was reached; then, the specimens were dried in air overnight at 80 °C in an oven. The resulting materials (∼10 mm disks) were directly used as binder-free electrodes for electrochemical testing.

To study the effect of the binder on the electrochemical performance, one material (CFP_430) was used to prepare a classical electrode: powder mixed with binder and carbon black. For this purpose, CFP_430 (90 wt%) was ground and mixed with polytetrafluoroethylene (PTFE) binder (5 wt%) and carbon black C65 (5 wt%) in ethanol solution at 80 °C until complete solvent evaporation; then, it was rolled, and self-standing electrodes were cut at the desired size. The preparation scheme for CFP_430 + PTFE + C65 is presented in Fig. S2.†

For comparison purpose, a reference electrode material based on a soft-salt template carbon powder and PTFE binder was used in this study (denoted KCl–T_ele). The electrode was synthesized according to the method reported previously on a soft-salt template employing the same soft-template (phenolic resin) and metal salt (KCl) as the CFP samples.^31^ The self-standing KCl–T_ele preparation was identical to the procedure described above for CFP_430 (Fig. S2†).

### 2.2. Physicochemical characterization of materials

The CFP features (if applicable also FP), including morphology, texture and surface chemistry, were characterized. The morphology of the binder-free electrodes was evaluated by an FEI Quanta 400 scanning electron microscope (SEM) with a high-resolution low-vacuum field emission gun (FEG). SEM micrographs were evaluated using ImageJ to obtain the fiber diameter and electrode thickness. The fiber diameter was measured for at least 100 fibers visible on the micrographs, and standard statistical analysis was performed to determine the standard deviation and mean values. The microscope was equipped with a JED 2300 detector for energy-dispersive X-ray spectroscopy (EDX) analysis. Several EDX acquisitions were recorded on large areas to determine the compositions of carbon filter papers; the average compositions are presented herein.

X-ray photoelectron spectroscopy (XPS) was used to study the surface chemical compositions of the materials (maximum analysis depth of 10 nm). The measurements were performed by using a VG Scienta SES 2002 spectrometer equipped with a monochromatic X-ray source (Al Kα = 1486.6 eV) and a VG Scienta XM780 monochromator.

The X-ray diffraction (XRD) patterns were recorded with a Bruker D8 Advanced diffractometer with flat-plate Bragg–Brentano *θ*–*θ* geometry equipped with a LynxEye XE-T high-resolution energy-dispersive one-dimensional (1D) detector (Cu Kα_1,2_). An angular range of 10–50° and step size of 0.01° (2*θ* scale) were used. The sample was laid on a Si waver sample holder, and its contribution was removed to better visualize the carbon diffraction peaks. Raman spectra were measured with a LabRAM BX40 spectrometer (Horiba Jobin-Yvon) equipped with a He–Ne excitation source (532 nm wavelength). Several spectra were acquired (mapping), and the average spectrum was used.

The textural properties of the materials were analyzed by two complementary gases, N_2_ at 77 K and CO_2_ at 273 K, using a Micromeritics ASAP 2020 device. Before analysis, the samples were outgassed on the degassing ports under secondary vacuum conditions for 12 h at 300 °C and for two additional hours on the analysis ports. Depending on the probe molecule used, the adsorption isotherms were recorded in the relative pressure (*P*/*P*_0_) ranges of 0–0.99 for N_2_ and 0–0.03 for CO_2_. The Brunauer–Emmett–Teller (BET) specific surface area was determined based on the linear fit in the relative pressure range of 0.01–0.05 for N_2_ (SSA__N_2__) and 0.01–0.03 for CO_2_ (SSA__CO_2__). The pore size distribution (PSD) was calculated from N_2_ and CO_2_ adsorption isotherms using the 2D nonlocal density functional theory (2D-NLDFT) standard slit model for carbon materials, which was conducted by using SAIEUS software.^34^ Moreover, the micropore volume *V*_micro_ was calculated in the *P*/*P*_0_ range of 10^−4^ to 10^−2^ using the Dubinin–Radushkevich equation for N_2_ and CO_2_. Furthermore, *V*_meso_ was calculated for the N_2_ adsorption branch as *V*_T_ − *V*_micro_, where *V*_T_ was obtained at 0.99 *P*/*P*_0_. The average diameter of the micropores (*L*_0_) was determined (<2 nm) for N_2_ and CO_2_ adsorbates, as reported elsewhere.^35^

The conductivity of the electrode material was recalculated from the resistance value recorded by electrochemical impedance spectroscopy (EIS) at 100 kHz in the Swagelok cell by considering the real electrode thickness.

### 2.3. Electrochemical characterization

Electrochemical investigations were performed in a Swagelok cell, which is a two-electrode cell with and without a reference electrode. Symmetric ECs were constructed using two identical electrodes separated by a glass fiber spacer with a thickness of 260 μm (Whatman GF/A). A saturated calomel electrode (SCE) was used as the reference electrode. Electrochemical tests in the voltage range from 0 to 0.8 V included cyclic voltammetry (CV) with various scan rates (1–100 mV s^−1^) and constant current charge/discharge (0.1–10 A g^−1^). Electrochemical impedance spectroscopy was conducted at 0 V in the frequency range of 1 mHz to 100 kHz with an amplitude of ±5 mV.

Rate handling was calculated by the percentage ratio between the specific capacitance values calculated at high (10 A g^−1^) and low current densities (0.1 A g^−1^) during constant current charge/discharge processes. Rate handling calculated from cyclic voltammetry is the ratio of specific capacitance reached at high (100 mV s^−1^) and low (1 mV s^−1^) scan rates, and the last rate handling addresses the ratio between specific capacitance calculated at high (1 Hz) and low (1 mHz) frequencies in impedance spectroscopy; thus, it is a metric related to the power response. All electrochemical parameters presented in the manuscript, were either calculated based on an active mass of electrode (in case of binder-free electrodes, it was the mass of the whole electrode disc) − specific capacitance (F g^−1^), or micropore volume or bulk density of electrode material − volumetric capacitance (F cm^−3^).

An EQCM (QCM922A, Seiko, Japan) study needed a binder-free electrode attachment to the quartz crystal resonator, which was achieved using conductive carbon DAG (Loctite, Henkel). Resonators with glued small (4 mm diameter) CFP samples were dried overnight at RT. Solid-state electrode attachment could influence the recorded resistance and charge propagation characteristics, causing them to differ from the results obtained in the Swagelok cell. To demonstrate the rigid coating requirement for Sauerbrey equation validation, the resistance change (Δ*R*) was recorded over cyclic voltammetry tests, and only experiments with a small variation, ±2%, were accepted for further data analysis (please see ESI Fig. S11†).^36,37^ The cell was filled with 0.4 mL of electrolyte, avoiding the presence of bubbles. The counter electrode was oversized stainless steel, and the reference electrode was an SCE. Cyclic voltammetry with a scan rate of 5 mV s^−1^ was applied in the potential window adjusted to oxidation–reduction reactions.

## 3. Results and discussion

### 3.1. Physicochemical characterization

**Fig. 2:**
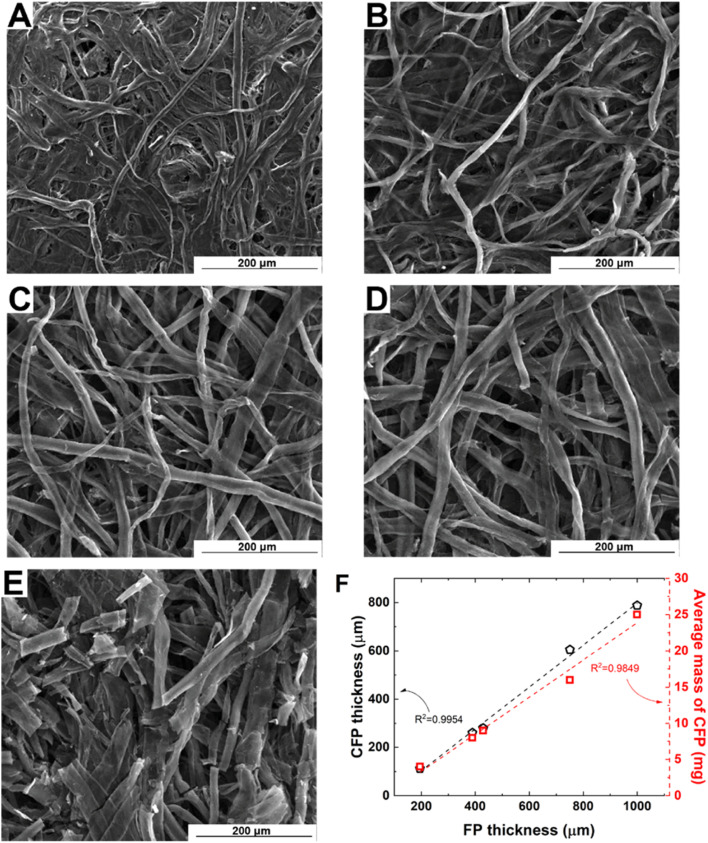
Morphology characterization of CFP electrodes: SEM micrographs (A) CFP_195; (B) CFP_390; (C) CFP_430; (D) CFP_700; (E) CFP_1000; and (F) correlation of precursor (FP) thickness and CFP electrode thickness and mass.

SEM images of the five CFP electrodes are presented in Fig. 2A–E. A complex network of randomly oriented entangled fibers and a macroporous structure were observed for all materials. The CFP_195 and CFP_390 structures seem much denser than the other binder-free electrodes due to their embedded structure (*i.e.*, the fibers seem to be glued together). This effect can result from the deposition of carbon from phenolic resin decomposition. For these materials, the impregnation process involving the carbon precursor can favor the reduced thickness or highly hydrophilic nature of the FPs. Conversely, the morphology of the CFP_1000 material is unique since the fibrous structure is locally broken, as presented in Fig. 2E; however, few entangled fibers can be observed. The average diameter of the fibers is determined to range between 10 μm (CFP_195) and 23 μm (CFP_1000), suggesting an increase with increasing film thickness, as seen in Fig. S3.†

Interestingly, in addition to having the same composition (Table S1 and Fig. S1†), the filter papers differ in precursor and synthesis routes. FP_195 is a qualitative cellulose filter paper. FP_390 is a low-ash qualitative filter paper that does not contain any additives. It is manufactured from high-quality cotton linters that have been treated to achieve a minimum alpha cellulose content of 98%. On the other hand, FP_430, FP_750, and FP_1000 are technical filter papers that contain an additive for better strength. These filter papers are made from super refined cellulose and have been specifically designed to have a high stability in long contact with water. Thus, samples FP_195 and FP_390 differ in the origin of the precursor and synthesis process from the other samples.

Regarding the binder-free electrodes, the heat treatment induces significantly smaller thicknesses (Table 1) than those of the raw filter papers (Table S1†). For example, for CFP_195, the thickness after pyrolysis decreases from 195 μm to 112 μm, while for CFP_1000, it decreases from 1000 μm to 788 μm. The evolution of the CFP thickness is directly proportional to the thicknesses of the initial FPs, with *R*^2^ = 0.99 (Fig. 2F). However, the thin electrode preserves 57% (CFP_197) of their initial thickness, differing from the thick electrodes (CFP_1000), which preserve approximately 78% of their initial thicknesses after pyrolysis. This finding can be due to electrode morphology, which seems very compact for thin electrodes relative to thick electrodes, as revealed by cross-section SEM images (Fig. S4†). Moreover, the masses of the obtained carbon electrodes are measured (Table 1), and they are directly proportional to the FP thickness (*R*^2^ = 0.98). However, if we calculate the mass shrinkage (FP to CFP) based on Table 1 and Table S1,† this is rather similar among all samples (40% for the thin electrode *vs.* 43% for the thick electrode). This indicates homogeneous phenolic resin impregnation through the FPs.

**Table 1:** Properties of CFP electrodes after synthesis

Label	Thickness, μm	Average mass, mg	Average fiber diameter, μm	Entangled	Embedded
CFP_195	112	4	10	YES	YES
CFP_390	261	8	11	YES	YES
CFP_430	280	9	17	YES	NO
CFP_750	605	16	19	YES	NO
CFP_1000	788	25	23	NO	NO

The material surface chemistry is evaluated by both EDX and XPS (Fig. S5†). Although there are some differences between the two techniques (wt% and at%), the results are in agreement for the elements identified: C, O and K. The EDX results reveal a carbon content between 83.6 wt% and 93.2 wt% for the CFP series, while for KCl–T_ele, an amount of 85.5 wt% is found. The amount of carbon in the materials may play an important role on electronic conductivity.^38^ The low amount of carbon in some materials implies that the O level is significant (Fig. S5A†), particularly for thick electrodes, such as CFP_750 (14.1 wt%) and CFP_1000 (14.4 wt%). The high oxygen content present in the CFP samples indicates high hydrophilicity, as proven by contact angle measurements (Fig. S5C†). This behavior is also favored by the high porosity. KCl–T_ele has a significant O level at 12 wt%, as well. In general, oxygen is found in functional groups, which can improve the wettability and redox reactions with electrolytes.^39^ However, a good balance between the oxygen and carbon contents must be ensured to not reduce electronic conductivity. Moreover, all the electrodes, except for CFP_390, present a low amount of K, accounting for a 2 wt% maximum (for CFP_750). The presence of K is confirmed for KCl–T_ele (1.9 wt%). The salt template washing step, which is performed under soft conditions (water instead of acid), can explain the randomly observed K traces in the structures of the materials.

**Fig. 3:**
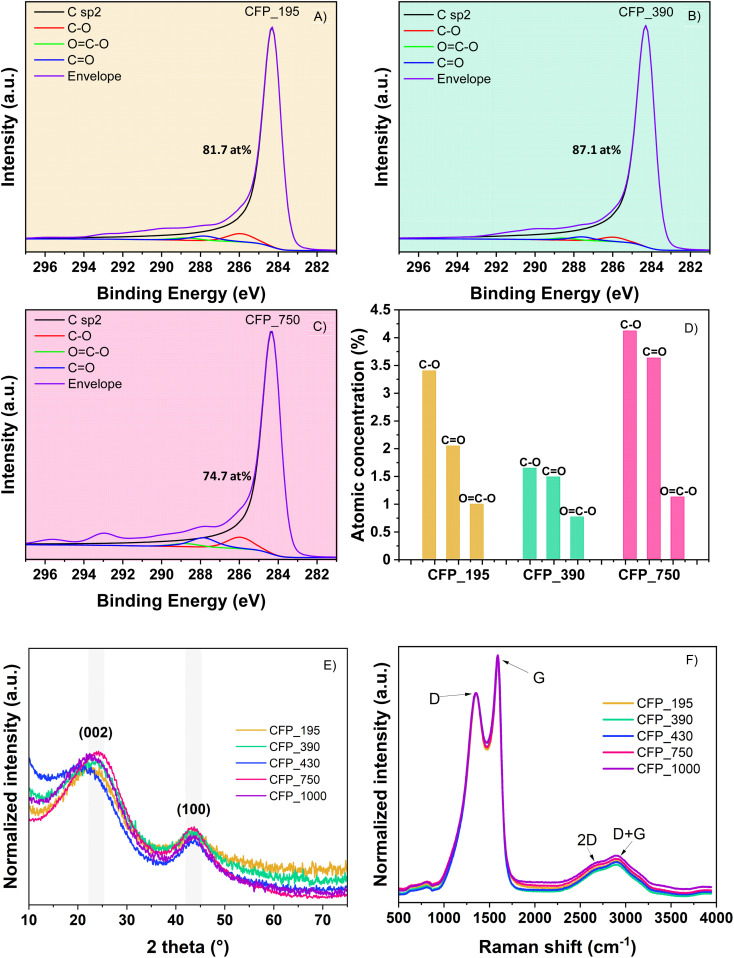
C 1s XPS high-resolution spectra of (A) CFP_195; (B) CFP_390; (C) CFP_750; and (D) concentration of oxygen-based functional groups of the same materials. Structural characterization of CFP materials: (E) XRD and (F) Raman spectra.

XPS is implied to provide additional insight into carbon structures and oxygen-based functionalities. Based on the above results, CFP_195, CFP_390 and CFP_750 were selected for XPS measurements (CFP_430 has a similar composition to CFP_390, while CFP_1000 is similar to CFP_750). The results in Fig. S5B† show similar trends for C composition, as revealed by EDX, *i.e.*, CFP_390 (94.8 at%) > CFP_195 (92.5 at%) > CFP_750 (86.8 at%). The high-resolution XPS C 1s peaks (Fig. 3A–C) show that the main contribution comes from the intense C sp^2^ peak and, to a small extent, from oxygen-based functional groups. Indeed, the oxygen surface composition varies in a manner opposite to that of carbon: CFP_750 (11.4 at%) > CFP_195 (6.9 at%) > CFP_390 (5.1 at%). Precisely, high-resolution XPS allows the identification of several types of oxygen functional groups, *i.e.*, ether (C–OR) and carbonyl (C=O) as predominant functional groups and carboxyl (COOR) and hydroxyl (OH) groups (Fig. 3D). Finally, small traces of K are found for CFP_195 and CFP_750 at 0.3 at% and 1.1 at%, respectively, suggesting the presence of K both on the surface and in the bulk of the sample.

XRD analyses show two peaks at 2*θ* = 22.3–24.2° and 43.5° corresponding to the (002) and (100) planes of the graphite structure. Their broad nature proves that CFP electrodes present a disordered-like structure (Fig. 3E). No visible peak from the KCl crystal structure is visible in the XRD spectra, which indicates good salt removal after the synthesis process. This is not exactly in agreement with the XPS results, where traces of KCl were identified and can be explained by the detection limit of the diffractometer (<2%).

In the Raman spectra (Fig. 3F), one can notice the D and G bands specific to the defects and graphitic domains in the materials. The *I*_D_/*I*_G_ ratio was found to be 1.67. In addition, broad 2D and D + G peaks are observed at high Raman shifts, >2500 cm^−1^. These results indicate that the materials present a disorder-like structure composed of amorphous and graphitic crystalline domains. This is the result of the interaction of the salt-template with the precursor. In our previous work,^40^ we found that LiCl can graphitize carbon at 900 °C due to its high cation–pi interaction with the aromatic rings of the phenolic resin, while KCl induced only local graphitization. Here, the mixture of cellulose/phenolic resin led to no obvious graphitization, most likely because of the large content of cellulose, which contains glucose rings that have lower reactivity with KCl at this temperature.

**Fig. 4:**
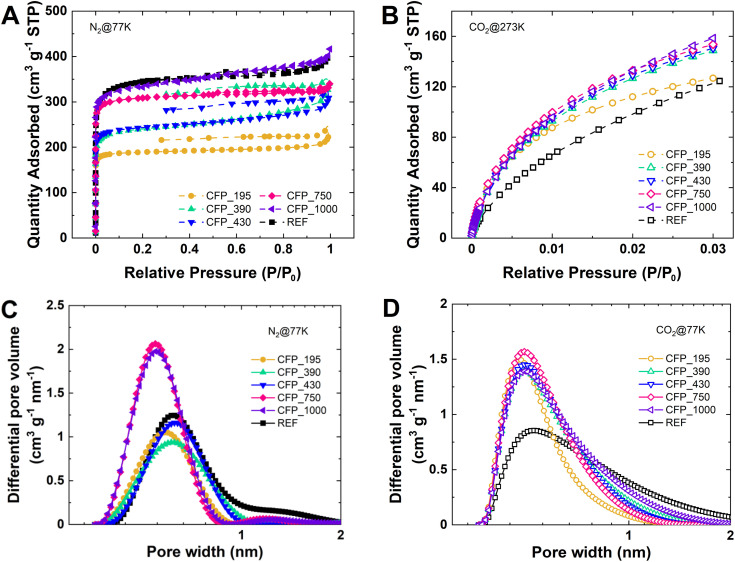
Texture characterization of CFP electrodes from N_2_ adsorption/desorption at 77 K and CO_2_ adsorption at 273 K. Isotherms: (A) N_2_ and (B) CO_2_. Pore size distribution: (C) N_2_ and (D) CO_2_.

The textural properties of the CFP binder-free electrodes and the KCl–T_ele reference are evaluated by N_2_ and CO_2_ (Fig. 4). The N_2_ adsorption/desorption isotherms are type I, characterized by a steep increase in N_2_ adsorbed at a low *P*/*P*_0_, which is followed by a plateau. At a high relative pressure (*P*/*P*_0_ ∼ 1), a small increase in adsorbed volume is observed for all materials, which is related to the presence of some mesopores from the entangled structure. This isotherm profile denotes mainly microporous materials and the presence of some mesopores. A particularity of the N_2_ isotherm is the irreversibility of the adsorption/desorption process indicated by the desorption branch that does not close, as seen for thin electrodes CFP_195, CFP_390 and CFP_430. This behavior reveals the presence of narrow pores with restricted access and/or particular shapes, such as the ink bottle shape.^41^

Notably, the N_2_ adsorbed volume increases as a function of material thickness for the CFP series, whereas for KCl–T_ele, the adsorbed volume is the highest, which is very similar to that of CFP_1000. The trend is confirmed by the specific surface area evolution (SSA__N_2__) presented in Table 2. CFP_195 has the lowest value at 762 m^2^ g^−1^, followed by CFP_390 at 950 m^2^ g^−1^ and CFP_430 at 970 m^2^ g^−1^. Next, CFP_750 has a surface area reaching 1237 m^2^ g^−1^, while CFP_1000 and KCl–T_ele exhibit the highest values at 1315 m^2^ g^−1^ and 1353 m^2^ g^−1^, respectively. In addition to SSA, the *V*_micro_ determined from the N_2_ adsorption increases from 0.29 cm^3^ g^−1^ for CFP_195 to 0.51 cm^3^ g^−1^ for CFP_1000. The volume of micropores found for KCl–T_ele is similar to that of the CFP_1000 material. Conversely, N_2_*V*_meso_ does not depend on CFP thickness and is rather small, with CFP_390 presenting the highest value (0.15 cm^3^ g^−1^).

**Table 2:** Texture characterization of CFP electrodes from N_2_ adsorption/desorption at 77 K and CO_2_ adsorption at 273 K

Electrode	SSA__N_2__, m^2^ g^−1^	*V*_micro_, cm^3^ g^−1^	*V*_meso_, cm^3^ g^−1^	*L*_0_, nm	SSA__CO_2__, m^2^ g^−1^	*V*_micro_, cm^3^ g^−1^	*L*_0_, nm
CFP_195	762	0.29	0.05	0.60	708	0.37	0.55
CFP_390	950	0.33	0.15	0.65	915	0.50	0.62
CFP_430	970	0.35	0.10	0.64	912	0.49	0.60
CFP_750	1237	0.49	0.02	0.58	908	0.47	0.58
CFP_1000	1315	0.51	0.10	0.59	1009	0.57	0.65
KCl–T_ele	1353	0.51	0.06	0.74	925	0.56	0.76

This phenomenon can be explained by the various impregnation mechanisms due to different initial electrode thicknesses, as all materials are easily soaked in water solutions. In thick electrodes (such as CFP_1000), the amount of salt template is high, leading to a microporous texture in the electrode bulk. However, for thin electrodes (such as CFP_195), the overall salt template volume within the electrode in the first synthesis step is smaller than that for thick electrodes, reducing the specific surface area. The resin impregnation is homogenous for both thin and thick electrodes, as shown by the SEM cross-section images of the CFP materials (Fig. S4†).

CO_2_ adsorption measurements were performed at 273 K for an in-depth characterization of the microporosity of the materials. Interestingly, we can observe that the SSA__CO_2__ values are high (between 671 m^2^ g^−1^ and 1009 m^2^ g^−1^) but slightly lower than their N_2_ counterparts, particularly for thick electrodes. This trend is in line with the N_2_ isotherms that show reversible desorption for thick electrodes and irreversible desorption for thin electrodes; thus, the presence of narrow pores is accessible by CO_2_. Therefore, these results indicate that the materials have a well-developed micro/ultramicroporous texture. Furthermore, the ultramicroporosity does not depend on the CFP thickness. SSA__CO_2__ increases from 708 m^2^ g^−1^ for CFP_195 to ∼915 m^2^ g^−1^ for CFP_390 and CFP_430, and it is slightly lower for CFP_750 (908 m^2^ g^−1^), while the highest value is obtained for CFP_1000 (1009 m^2^ g^−1^). A random evolution is observed for the *V*_micro_ obtained from CO_2_ adsorption *vs.* material thickness, which varies between 0.37 and 0.57 cm^3^ g^−1^. Nevertheless, KCl–T_ele exhibits close textural properties as CFP_1000 when CO_2_ is used (925 m^2^ g^−1^ with a *V*_micro_ of 0.56 cm^3^ g^−1^).

Concerning the average micropore size (*L*_0_), similar values can be found for the same material when using either N_2_ or CO_2_ adsorbate (Table 2, Fig. 4C and D). Among the materials, *L*_0_ clearly does not depend on the CFP thickness and has a size that varies between 0.55 and 0.65 nm; the same salt template (KCl) is used to create microporosity. KCl–T_ele has the highest micropore size for N_2_ (0.74 nm) and CO_2_ (0.76 nm). According to the literature, a pore size of approximately 0.7 nm is perfectly suitable for the formation of an electric double layer.^42–44^

### 3.2. Electrochemical characterization

CFP samples are tested in aqueous EC with 1 mol L^−1^ KOH electrolyte. The choice of electrolyte is motivated by our previous finding that the cation electrolyte that matches the salt-template used during synthesis (KOH that matches the KCl salt-template) shows the best performance in EC applications.^31^ As discussed in the physicochemical characterization section, samples are synthesized in the same manner with different FP precursors. Consequently, the samples exhibit different physicochemical properties (texture, chemical composition, thickness and morphology). These differences translate into their electrochemical performance. For comparison, reference soft-salt templated carbon (KCl–T_ele) is added to the plots with permission.^31^ Notably, the addition of binder (5 wt% PTFE) is needed to form a self-standing electrode from KCl soft-salt templated carbon.

**Fig. 5:**
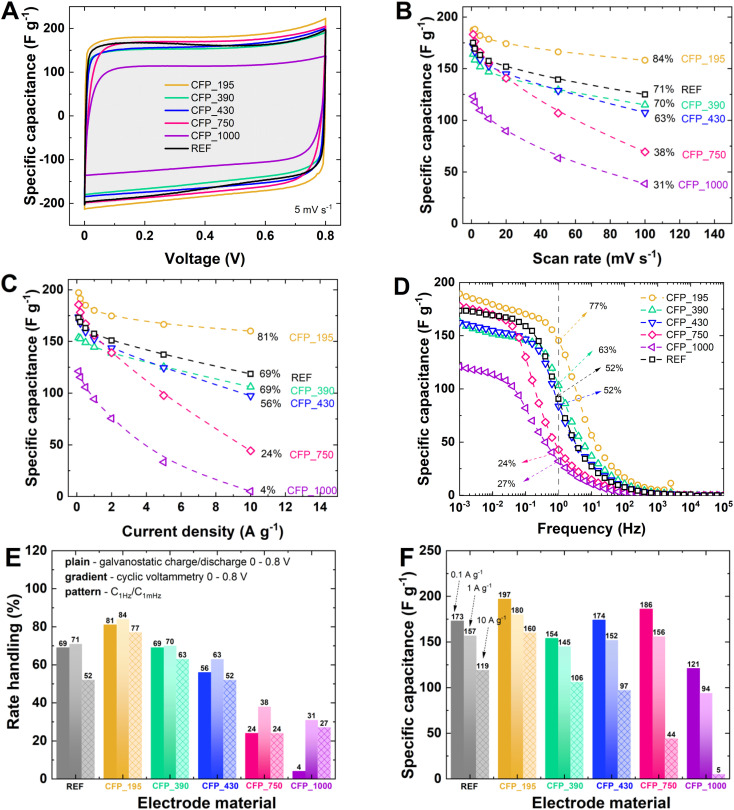
Electrochemical characterization of CFP electrodes with 1 mol L^−1^ KOH: (A) cyclic voltammograms at 5 mV s^−1^; specific capacitance *vs.*: (B) scan rate; (C) current density; and (D) frequency. Electrochemical performance metrics as functions of the electrode types: (E) rate handling from galvanostatic charge/discharge, cyclic voltammetry, and electrochemical impedance spectroscopy. (F) Specific capacitance value calculated for current densities of 0.1, 1 and 10 A g^−1^.

Fig. 5 presents the electrochemical characteristics and a comparison between all CFP samples with KCl–T_ele. All samples are characterized by good charge propagation that is visible in a rectangular CV shape with a dominant capacitive storage mechanism (Fig. 5A). The specific capacitance behavior at different scan rates clearly shows that sample CFP_195 exceeds the performance of KCl–T_ele (capacitance retention 84% *vs.* 71%; Fig. 5B). Similar comparisons were performed for constant current charge/discharge (Fig. S6A†), which proves the findings from cyclic voltammetry studies. CFP_195 has the best rate capability (81%), while CFP_1000 has the poorest rate capability (4% at 10 A g^−1^). Sample CFP_390 behaves like KCl–T_ele, qualitatively and quantitatively (rate handling at the level of 69%; Fig. 5C). Impedance spectroscopy conducted at 0 V (Fig. 5D and S6B†) shows good charge propagation with increasing frequency with rate handling at 77% for CFP_195. Clearly, electrochemical performance lies in the trend of electrode thickness; that is, the thinner the electrode is, the higher the capacitance and rate handling levels. This trend proves that CFP samples do not differ from standard powder carbon when synthesized with the same soft-salt template protocol (KCl–T_ele). Therefore, self-standing electrodes that are sustainable (not F-containing) and easy to handle can become a successful replacement of standard electrodes with a fluorinated polymer binder additive.

Rate handling is calculated from different electrochemical tests: cyclic voltammetry (*C*_ele_ @ 100 mV s^−1^/*C*_ele_ at 1 mV s^−1^), galvanostatic (*C*_ele_ at 10 A g^−1^/*C*_ele_ at 0.1 A g^−1^) and impedance spectroscopy (*C*_ele_ at 1 Hz/*C*_ele_ at 1 mHz). Fig. 5E clearly indicates that increasing the thickness of the CFP electrode decreases the amount of charge stored at high loads. Independent of the electrochemical technique applied, this behavior is observed for all CFP samples. Among all materials, CFP_195 and CFP_390 are the most promising for EC applications. CFP_430 does not deviate drastically and is near the rate handling values of KCl–T_ele. Samples CFP_750 and CFP_1000 exhibit the poorest electrochemical performance. The very low-rate handling of <50% results from poor electrochemical behavior under high loads. Fig. 5F shows that the specific capacitance values recorded during charge/discharge with constant currents of 0.1 and 1 A g^−1^ do not distinguish samples CFP_750 and CFP_1000 as the poorest specimens. However, the specific capacitance recorded at a high current load (10 A g^−1^) is drastically low, directly affecting the low-rate handling value (Fig. 5E). This finding can be explained by the fact that at low current regimes, there is sufficient time for electrolyte diffusion through the well-developed porosity, even if the electrode thickness is high. However, at high currents, the rate capability is limited by the high thickness and long diffusion pathways, impeding the fast adsorption/desorption rates of electrolyte ions through the material.

**Fig. 6:**
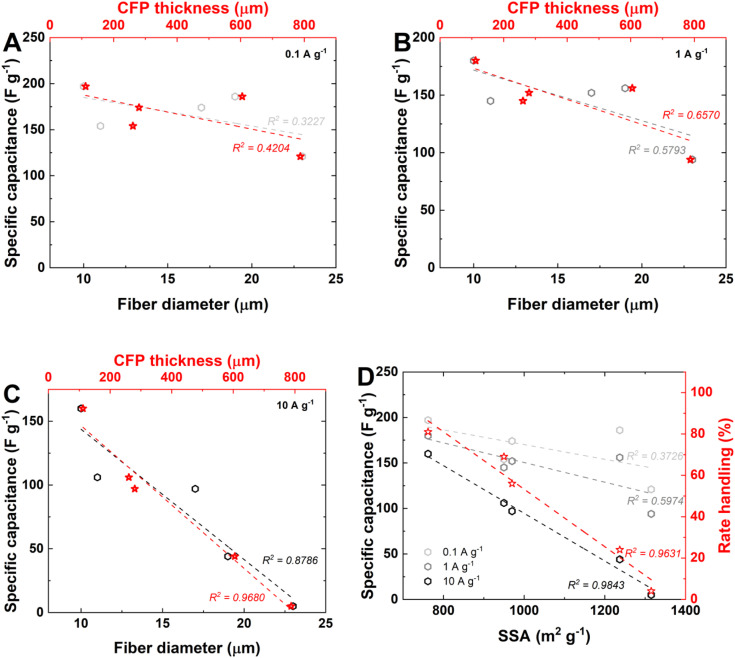
Correlation of physicochemical parameters with electrochemical performance: specific capacitance *vs.* fiber diameter and CFP thickness: (A) 0.1 A g^−1^; (B) 1 A g^−1^; and (C) 10 A g^−1^. (D) Specific capacitance and rate handling *vs.* SSA__N_2__.

To understand which physicochemical features are key to determining the electrochemical performance of CFP electrodes, Fig. 6 is proposed. Fig. 6A–C present the correlations of the specific capacitances at various current densities *vs.* the electrode thicknesses and fiber diameters. The *R*^2^ is presented for both correlations: *vs.* fiber diameter (presented in black) and *vs.* electrode thickness (presented in red). The increase in charge/discharge current density improves the linear correlation (*R*^2^ > 0.95), following the trend of the specific capacitance value decreasing with increasing CFP thickness. The increase in *R*^2^ from 0.42 at 0.1 A g^−1^ to 0.65 at 1.0 A g^−1^ and 0.97 at 10 A g^−1^ indicates that at a high current density, the electrode thickness becomes the main factor affecting the rate capability. The same tendency is seen with the fiber diameter, suggesting that increases in electrode thickness and fiber govern the rate capability at high regimes.

Fig. 6D presents a combination of the specific capacitance calculated at 10 A g^−1^ (represented in black) with the rate handling from galvanostatic charge/discharge (presented in red) *vs.* SSA__N_2__ of electrodes. This correlation is the highest (*R*^2^ > 0.96). Interestingly, high SSA__N_2__ values do not lead to high specific capacitances at this particular current rate. This result proves that the accessible surface area is different from the specific surface area. For example, CFP_1000 has the highest specific surface area (Table 2) and the lowest capacitance at high current rates. The binder-free electrodes are homogenous, and their fibrous morphologies should enable high ion mobility within the electrode bulk; at high thicknesses, ion diffusion might be impeded, as for CFP-1000. In contrast, for the thin electrode CFP_195, the electrode bulk is fully accessible for ions to form an electrical double layer, thus leading to the best electrochemical performance, even if the electrode has the lowest SSA__N_2__. Therefore, at high specific capacitance values (160 F g^−1^ @ 0.1 A g^−1^) and very high-rate handling values, no spatial hindrance occurs within the electrode bulk (81% from constant current charge/discharge), as exhibited by CFP_195.

Rate handling is clearly linearly correlated with fiber diameter and CFP thickness (*R*^2^ > 0.92; Fig. S7A†). The higher the thickness and fiber diameter are, the lower the rate handling value, which is in line with the findings of other works.^45–47^ This result suggests that ions are likely to leave the electrode/electrolyte interface during discharging for the CFP_750 and CFP_1000 electrodes. We try to correlate the specific capacity and/or rate handling with the O content (Fig. S7B†) and CFP conductivity values (Fig. S7C†); unfortunately, these correlations do not provide strong support for the interplay of these parameters. However, some general information can still be extracted. A high oxygen content reduces the specific capacitance and rate handling values. In particular, an oxygen level above 10 wt% is not beneficial for electrodes in aqueous EC. Below 10 wt% oxygen, there is no strict trend, as such an amount does not deteriorate the performance of the CFP electrode. Interestingly, increasing the conductivity of the CFP electrodes decreases the specific capacitance and rate handling values. Conductivity can be correlated with the structural properties of the electrode material; as discussed in the literature,^24^ it is not directly correlated with electrochemical performance. Most likely, samples with high conductivity tend to strongly adsorb ions at the electrode/electrolyte interface, reducing their mobility in the bulk.

**Fig. 7:**
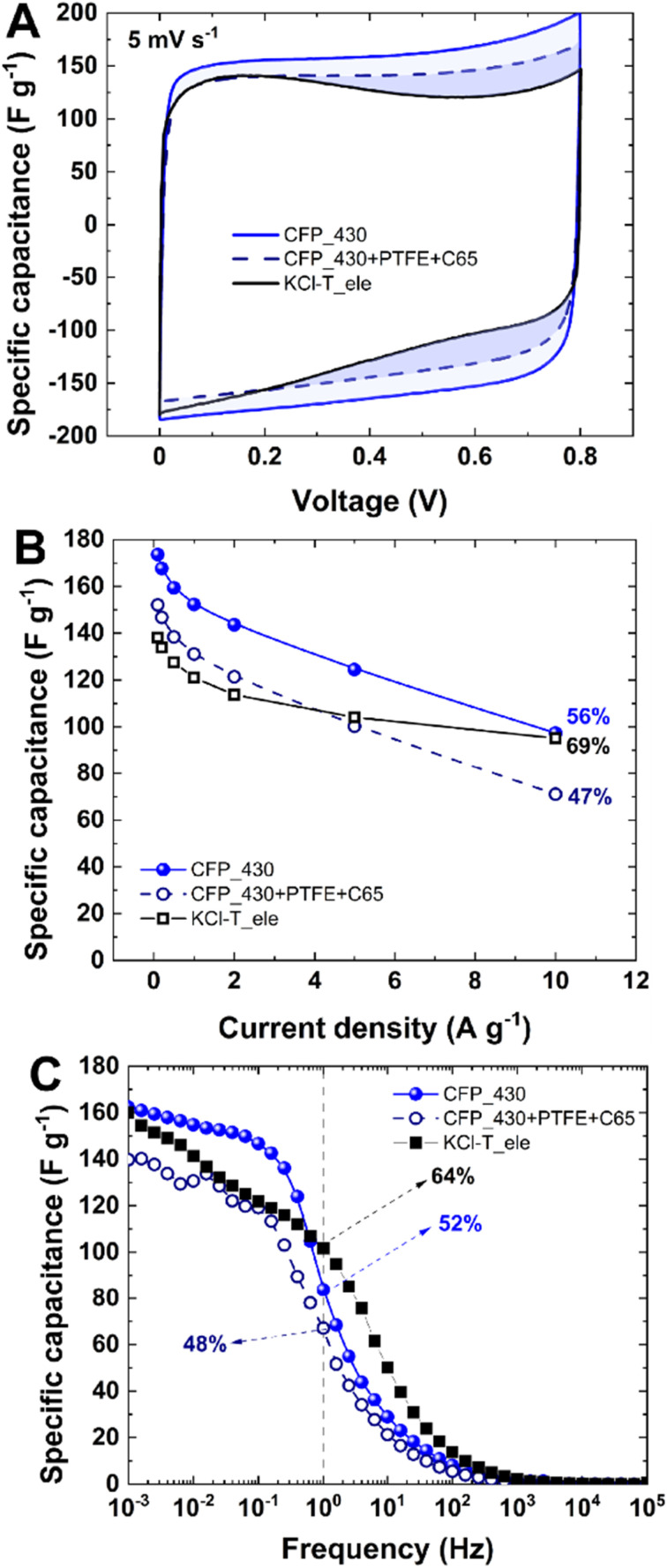
Comparison of the binder-free CFP_430 electrode with the CFP_430 powder-like electrode containing binder and carbon black (90 : 5 : 5) and reference KCl–T_ele material according to ref. 31: (A) cyclic voltammetry at 5 mV s^−1^; (B) specific capacitance *vs.* current density; and (C) specific capacitance *vs.* frequency.

To study the influences of binders on electrode performance in EC, three samples are compared (Fig. 7): binder-free CFP_430, ground CFP_430 + 5 wt% PTFE + 5 wt% C65 (denoted CFP_430 + PTFE + C65) and ground KCl–T + 5 wt% PTFE + 5 wt% C65 (denoted KCl–T_ele, data taken with permission from ref. 31). Sample CFP_430 is selected based on the performance being most similar to that of the reference KCl–T_ele. Therefore, it can be assumed that the presence/absence of the binder affects the EC performance of this comparison. From the cyclic voltammetry profile at 5 mV s^−1^ (Fig. 7A), the binder-free electrode CFP_430 does not exhibit the limitations of charge storage (rectangular CV) at elevated voltages. Samples containing PTFE binder exhibit butterfly shaped CV, indicating some distortion of charge accumulation at elevated voltages (>0.4 V). This trend is more pronounced for the KCl–T_ele sample than for CFP_430 + PTFE + C65. The rate handling of galvanostatic charge/discharge is slightly lower for the CFP_430 samples than for KCl–T; this trend is in accordance with the data presented in Fig. 5. The CFP_430 + PTFE + C65 sample shows specific capacitance and rate handling values that are *ca.* 10% smaller than those of the binder-free CFP_430 electrode in the galvanostatic charge/discharge test.

Specific capacitance *vs.* frequency shows that binder-free CFP_430 is the most stable specimen in the low-frequency region (up to 200 mHz), exhibiting purely capacitive behavior. KCl–T_ele does not maintain the charge accumulated at the electrode/electrolyte interface after fast charging/discharging. The CFP_430 + PTFE + C65 sample reveals the most unstable performance with a low-rate handling value of <50% for C_1 Hz_/C_100 kHz_. This study proves that the synthesis of binder-free electrodes should be a key research target for the carbon society, as binder additives deteriorate electrode performance and destabilize EC behavior. It is feasible to obtain a binder-free porous electrode material with a developed surface area with high accessibility and good mechanical integrity. Binder-free samples exhibit volumetric capacitance comparable to that of other binder-free porous carbons in the range of 200–600 F cm^−3^. The volumetric capacitance based on *V*_micro_ is linear for increasing CFP thickness (Fig. S7D†), and the trend is preserved at different current densities – 0.1, 1 and 10 A g^−1^. Furthermore, different sample geometries and lengths can be achieved, *i.e.*, a large rectangular sample (size ∼ 5 cm) and small spherical sample (size ∼ 0.4 cm), as shown in Fig. S8.† This highlights the versatility of the synthesis process to obtain tuned electrodes to satisfy diverse applications and large-scale implementation.

**Fig. 8:**
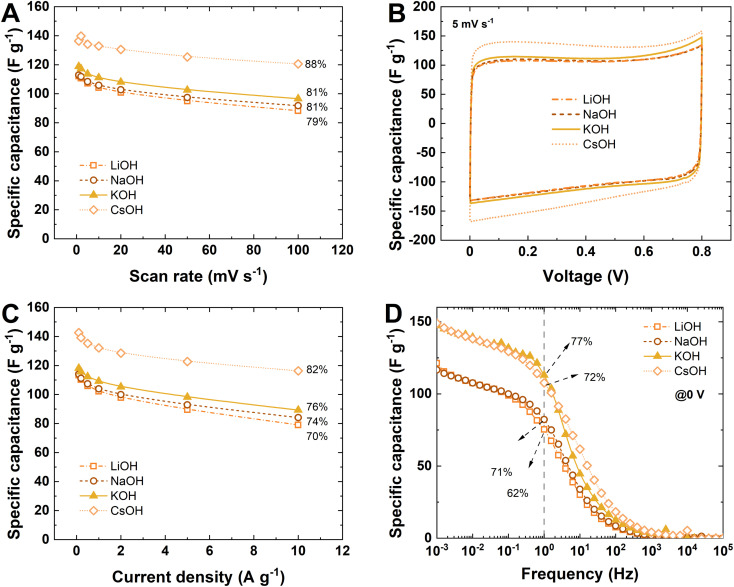
Electrochemical characterization of the CFP_195 electrode with 1 mol L^−1^ MOH (M = Li^+^, Na^+^, K^+^, and Cs^+^): (A) specific capacitance *vs.* scan rate; (B) cyclic voltammograms at 5 mV s^−1^; (C) specific capacitance *vs.* current density; and (D) specific capacitance *vs.* frequency.

To study the pairing of specific electrode/electrolyte ions, a set of 1.0 mol L^−1^ alkali-metal hydroxide (MOH) solutions (where M = Li^+^, Na^+^, K^+^, and Cs^+^) are used as electrolytes in EC according to the procedure described in ref. 31. For this purpose, the most promising binder-free CFP electrode is selected—CFP_195—which exhibits the highest specific capacitance and rate handling values among all CFP samples (Fig. 8). According to the data published for soft-salt-templated carbon KCl–T_ele,^31^ electrode/electrolyte matching is observed when considering cations (Li^+^, Na^+^ and K^+^). This finding suggests that the best performance regarding specific capacitance and rate handling should be observed in all electrochemical techniques tested for CFP_195 combined with a 1 mol L^−1^ KOH electrolyte; the salt template used during electrode synthesis is KCl. Interestingly, we extend this research by applying 1 mol L^−1^ CsOH as the electrolyte, and we observe that the performance of CFP-based EC is greatly enhanced, showing a *ca.* 15% increase in specific capacitance and a *ca.* 6% increase in rate handling. This electrolyte is selected based on very promising results obtained from soft-salt templated materials, where the electrolyte properties surpass the electrode texture/electrolyte ion matching.^31^ However, from an application point of view, this electrolyte (1 mol L^−1^ CsOH) is not recommended due to the high capital expenditure cost. On a laboratory scale, the cost of the CsOH electrolyte (1 mol L^−1^, 250 μL) is ∼200× more expensive than that of KOH (1 mol L^−1^, 250 μL) (prices verified at Sigma Aldrich on 12.04.2023). From this viewpoint, a 15% increase in specific capacitance is not worth the 150-fold increase in capital cost.

The electrochemical performance of the CFP_195 electrode with 1 mol L^−1^ hydroxide solution MOH (where M = Li^+^, Na^+^ and K^+^) follows trend electrode/electrolyte matching. The best performance is observed for the EC composed of CFP_195 with 1 mol L^−1^ KOH electrolyte. The CFP_195 electrodes were tested with 1 mol L^−1^ KOH in a two-electrode system with a reference electrode to observe the behavior of each separately. Fig. S9A and B† presents data recorded during galvanostatic charge/discharge at 1 A g^−1^ and the corresponding CV profiles (Fig. S9C and D† for 0.1 A g^−1^). The positive electrode operates in a high potential range, even though the OH^−^ ions are much smaller and more mobile than K^+^. If no redox contribution is presumed, the electrode potential should be similar. This discrepancy between the positive and negative electrodes suggests a difference in the charge storage mechanism due to a possible faradaic contribution from K^+^ insertion into carbon pores due to electrolyte ion/electrode pore matching. A high potential range signifies that the electrode is characterized by a low capacitance; in this case, the positive charge accumulated at the electrode/electrolyte interface is small. This trend is correlated with this high K^+^ cation affinity to carbon, matching the salt template used during synthesis. The overall EC charge/discharge profile is characterized by high efficiencies, with a coulombic efficiency of 99% and an energetic efficiency of 90% at 1 A g^−1^ and a coulombic efficiency of 92% and an energetic efficiency of 77% at 0.1 A g^−1^, indicating a pseudocapacitance contribution rather than a faradaic contribution.

To verify this statement, EQCM studies were conducted for 1 mol L^−1^ and 0.1 mol L^−1^ KOH solutions in contact with the CFP_195 electrode (Fig. S10†). The Swagelok and EQCM cells operate within a similar potential window for both electrodes (Fig. S10A†) with 1 mol L^−1^ KOH. Both concentrations of electrolytes (Fig. S10B and C†)—1 and 0.1 mol L^−1^—show the same charge storage mechanism. Interestingly, for the potential range resembling negative electrode operation, the normalized mass change decreases, while the potential decreases toward increasingly negative values. This finding indicates that the desorption process is correlated with the reduction reaction.^48^ This phenomenon shows that inserted K^+^ ions need to repel water molecules or OH^−^ ions that already occupy the pore volume/electrode structure. Therefore, a complex charge storage mechanism can result in a narrow potential window for a negative electrode.

**Fig. 9:**
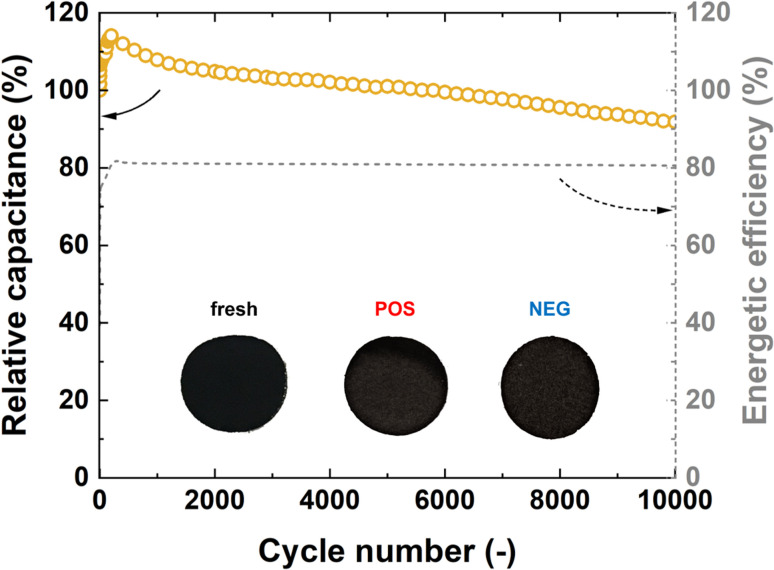
Cyclability test CFP_195 with 1 M KOH: relative capacitance recorded over 10 000 cycles at 1 A g^−1^ with energetic efficiency; inset: photo of electrodes before assembly (fresh) and after cycling test in Swagelok cell (POS and NEG).

Long-term performance studies were conducted for 10 000 cycles at constant current charge/discharge with a current density of 1 A g^−1^, as shown in Fig. 9. Sample CFP_195 was evaluated at the macroscale before and after operation (positive and negative electrodes) in terms of its mechanical integrity. The cyclability test was successful, with 90% capacitance retention and stable energetic efficiency of charging/discharging cycles over the whole experimental time.

This test proves the very good electrochemical properties of binder-free electrode materials and their high mechanical stability/integrity.

The last important characterization of CFP materials in ECs is their comparison with other binder-free electrode materials reported in the literature (Fig. S12†). We can see that CFP materials present average capacitance at low current densities compared to the literature papers; however, the capacitance retention of CFP at high current densities is very well preserved, whereas the other works do not provide such important data.

## 4. Conclusions

Binder-free porous carbon electrodes were successfully synthesized using a combined impregnation and soft-salt template method on filter papers. For these materials, no additional binder was needed to form a self-standing electrode. Therefore, electrochemical performance was directly linked to the electrode physico-chemical properties. In particular, the impact of filter paper thickness, in the range of ∼200 to 1000 μm, was studied. This investigation allowed us to obtain carbon filter paper electrodes with thicknesses linearly tuned by the initial thickness of the filter paper. The CFP porosity and oxygen surface functionalities increased with increasing electrode thickness. This enhancement favored ion adsorption and redox reactions, boosting the performance; however, increasing the electrode thickness and fiber diameter reduced the performance. This trend was significantly accentuated at high regimes, where these parameters became the key factors governing the performance. Thus, a linear correlation between the electrode thickness/fiber and the capacitance at 10 A g^−1^ was evidenced. The CFP_195 and CFP_390 electrodes exceeded the performance of the binder-built reference KCl–T_ele (regarding specific capacitance and rate handling) in various electrochemical tests (cyclic voltammetry, galvanostatic charge/discharge, and impedance spectroscopy). This finding indicated that the ion accessibility and optimal thickness of the electrode were crucial for satisfactory EC operation (specific capacitance at 5 mV s^−1^ with 1 mol L^−1^ KOH > 150 F g^−1^ and rate handling from galvanostatic tests ≥69%). In addition, electrode–electrolyte cation matching was proven with a series of alkali-metal hydroxide solutions. The cations present in the electrolyte solution matched the cations used during the soft-salt template synthesis of carbon materials. Complex charge/discharge behavior for the CFP_195 electrode was observed with a KOH electrolytic solution (0.1 and 1 mol L^−1^), showing a plausible potassium insertion process or reduction in carbon material within the negative polarization range. This work demonstrated the successful preparation of binder-free electrodes, providing better performance than that of the binder-electrode counterpart and reducing the possible environmental impacts of fluoride-based binders.

## Conflicts of interest

There are no conflicts to declare.

## Supplementary Material

TA-012-D3TA04971J-s001
