# Supplementary material for: Synthesis and performance of binder-free porous carbon electrodes in electrochemical capacitors
Source: J Mater Chem A Mater. 2024 Feb 14;12(11):6412–25. doi: 10.1039/d3ta04971j (PMC10929587; doi:10.1039/d3ta04971j)
Supplement: TA-012-D3TA04971J-s001 [file TA-012-D3TA04971J-s001.pdf]

Electronic Supplementary Material (ESI) for Journal of Materials Chemistry A.
This journal is © The Royal Society of Chemistry 2024

Supporting Information

Synthesis and performance of binder-free porous carbon electrodes in electrochemical capacitors

Anetta Platek-Mielczarek[a,b], Adrian Beda[c,d], Krzysztof Fic[a]*, Camelia Matei Ghimbeu[c,d,e]*

[a] Poznan University of Technology, Institute of Chemistry and Technical Electrochemistry, Berdychowo 4, 60–965 Poznan, Poland

[b] Laboratory for Multiphase Thermofluidics and Surface Nanoengineering, Department of Mechanical and Process Engineering, ETH Zurich, Sonneggstrasse 3, Zurich, Switzerland

[c] Université de Haute-Alsace, Institut de Science des Matériaux de Mulhouse (IS2M), CNRS UMR 7361, F-68100, Mulhouse, France

[d] Université de Strasbourg, F-67081, Strasbourg, France

[e] Réseau sur le Stockage Electrochimique de l'Energie (RS2E), CNRS FR3459, 33 Rue Saint Leu, 80039, Amiens Cedex, France

*Corresponding authors:

krzysztof.fic@put.poznan.pl, camelia.ghimbeu@uha.fr

Table S1. Properties of FP before synthesis as provided by the supplier and chemical composition measured using EDX.

	Physical properties						EDX analysis	
**Label**	**Name and supplier**	**Mass of circular sample 12mm diameter, mg**	**Thickness, µm**	**Filtration time, s**	**Surface weight, g m^{-2}**	**Retention range, µm**	**C, wt.%**	**O, wt.%**
**FP_195**	Rotilabo TYPE 15A	10.1	195	180	84	2-3	46.2	53.8
**FP_390**	Whatman Grade 3	21.9	390	325	185	6	46.0	53.9
**FP_430**	Whatman Grade 2589a	31.1	430	60	200	6-12	46.3	53.7
**FP_750**	Whatman Grade 2589c	43.2	750	160	400	4-8	45.9	54.1
**FP_1000**	Whatman Grade 2589d	58.5	1000	235	500	2-6	46.5	53.5

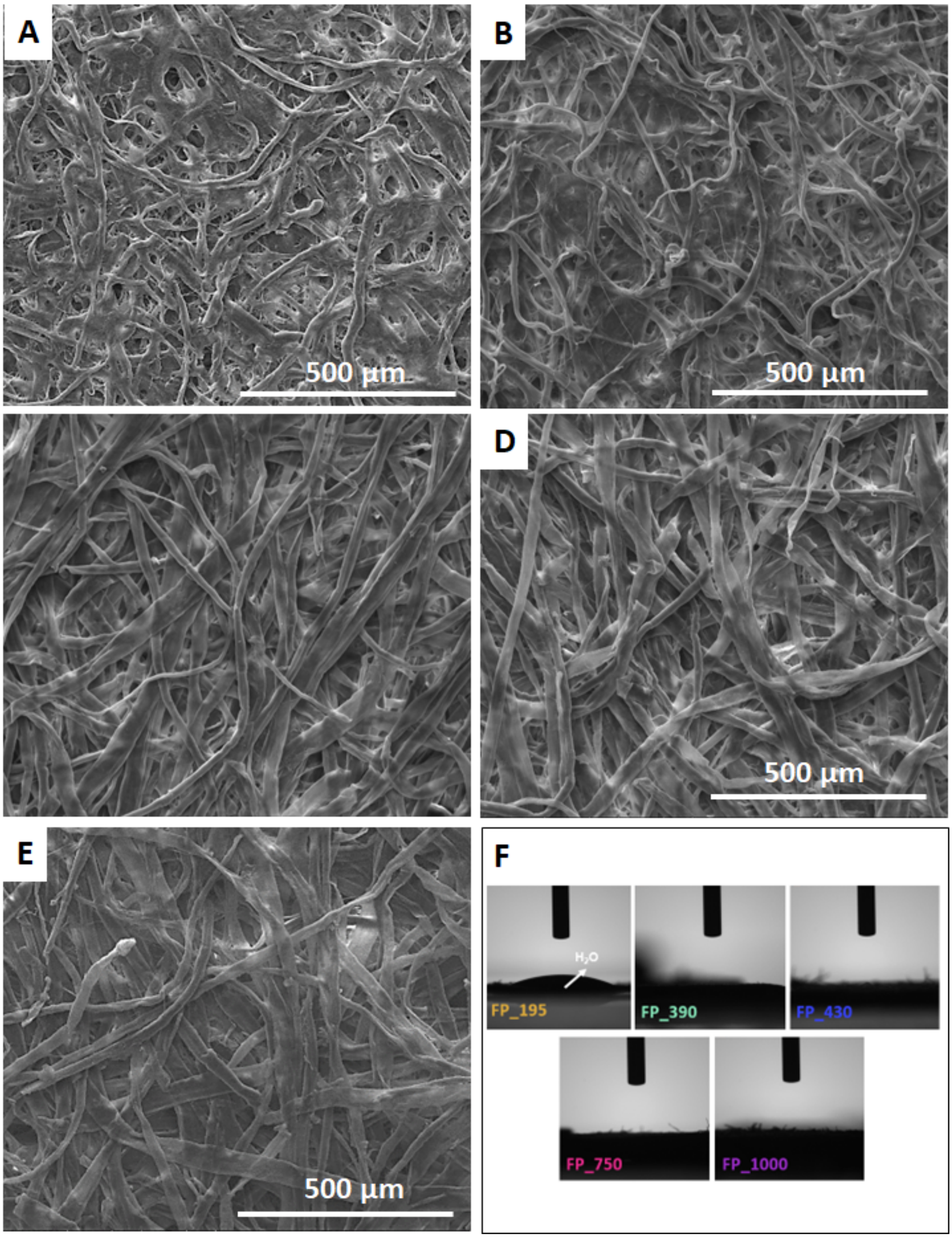

Figure S1. Characterization of FP: SEM micrographs A) FP_195; B) FP_390; C) FP_430; D) FP_750; E) FP_1000. Contact angle measurement using sessile drop analysis with water: F) all FP samples.

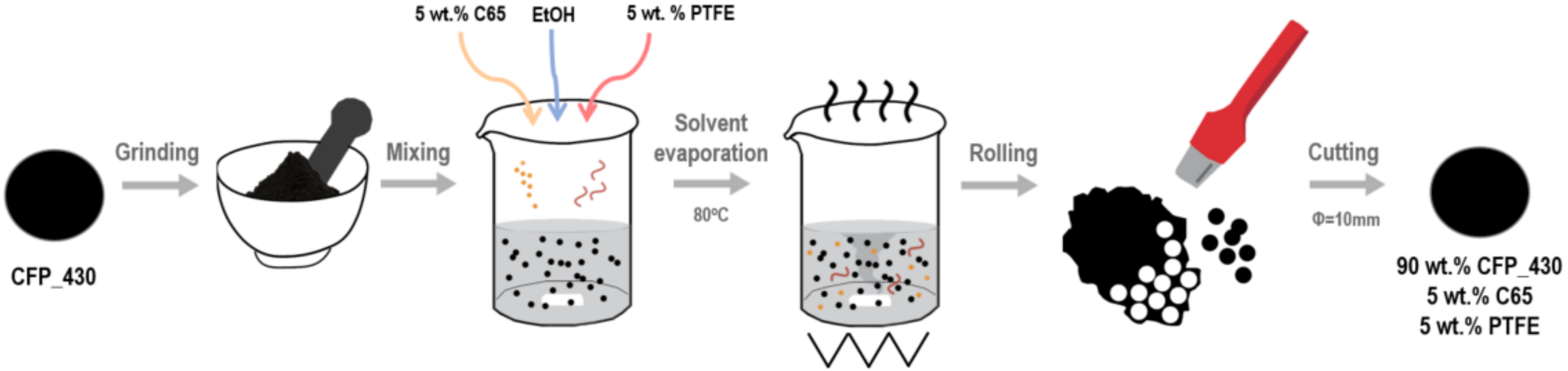

Figure S2. Scheme of using CFP electrodes as an active material in electrode composite (90wt.% AC, 5wt.% PTFE, 5 wt.% C65).

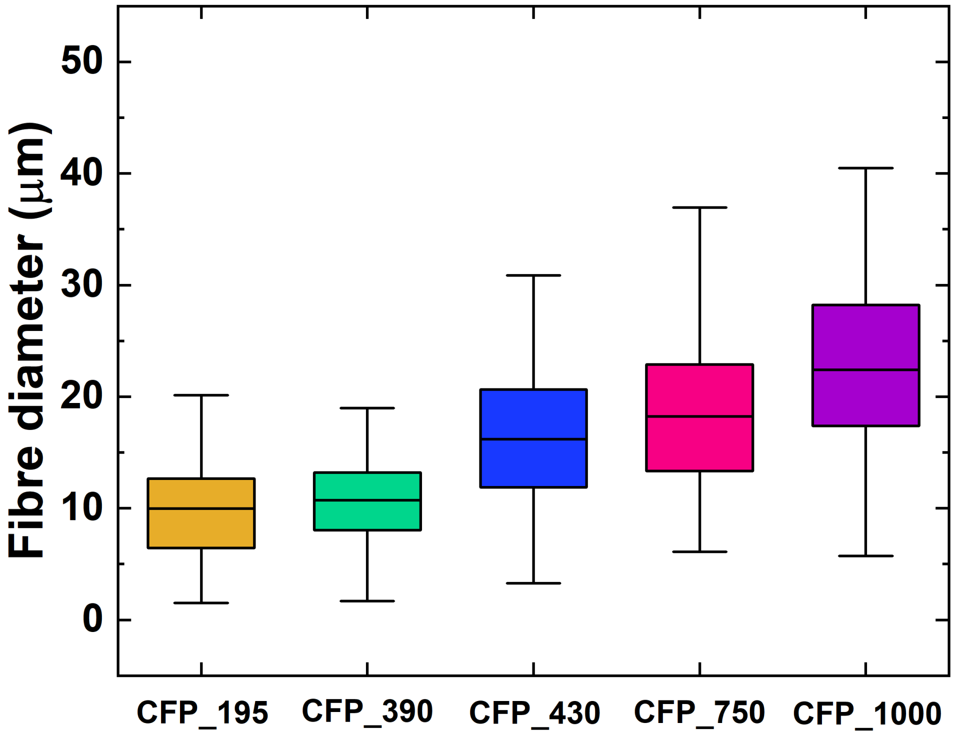

Figure S3. Analysis of fiber diameter from SEM micrographs represented for all CFP samples with standard deviation. Mean value is represented in a line within a bar.

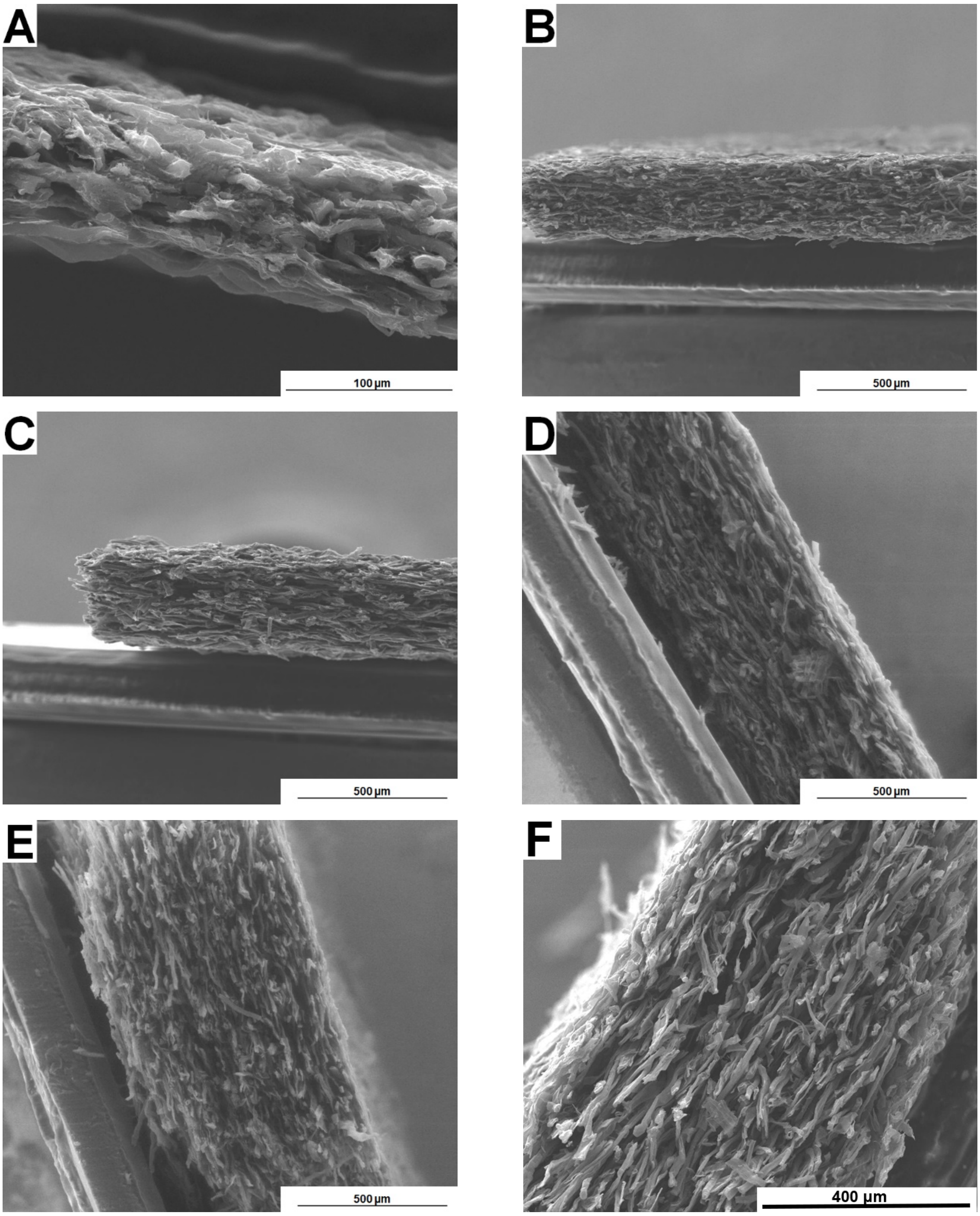

Figure S4. Cross sectional SEM micrographs of CFP electrodes: A) CFP_195; B) CFP_390; C) CFP_430; D) CFP_700; E) and F) CFP_1000 with two magnifications.

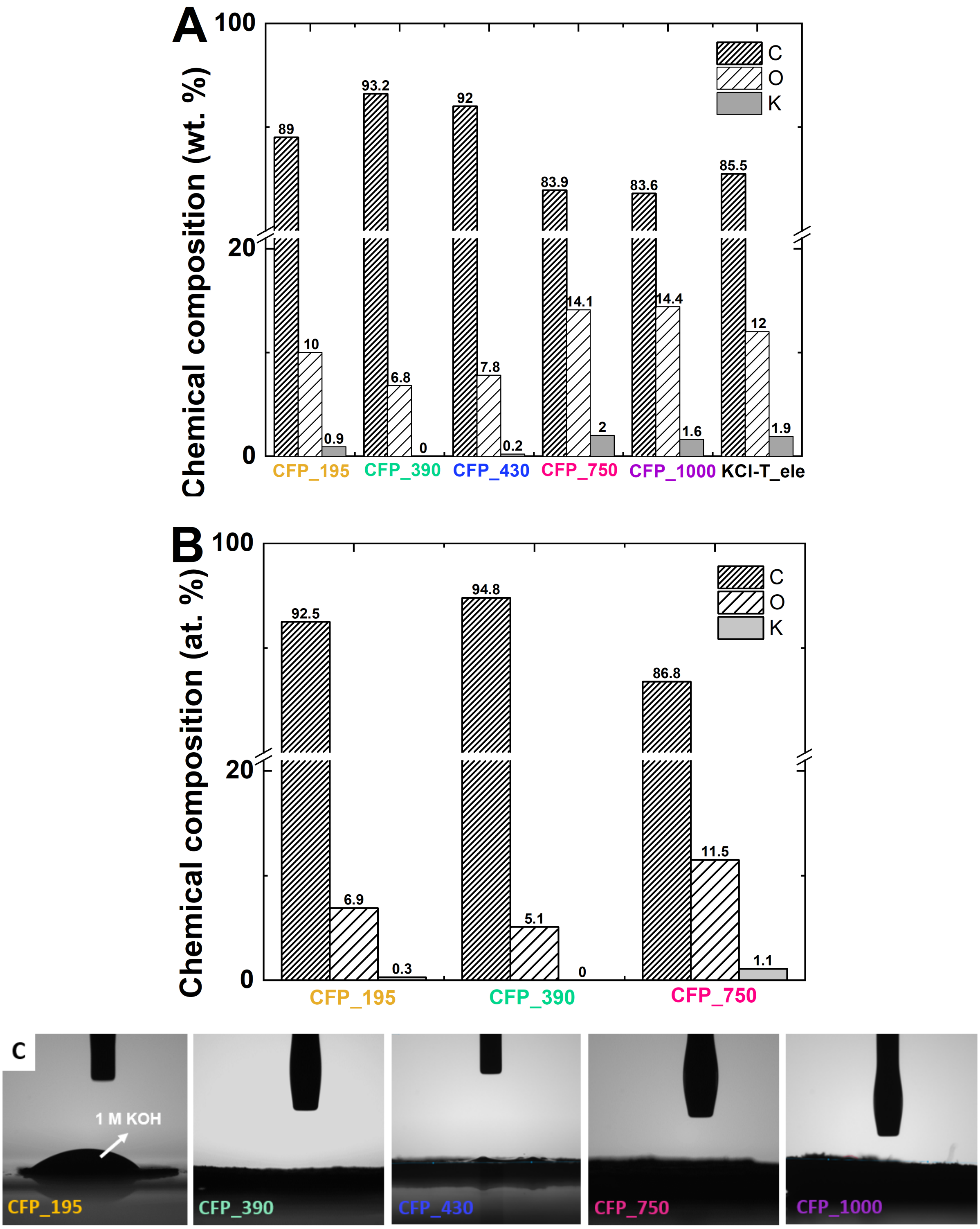

Figure S5. Surface chemical composition: A) from EDX for all CFP electrodes and KCl-T_ele; B) from XPS for CFP_195, CFP_390 and CFP_750 electrode; C) wettability tests using sessile drop measurement for CFP materials with 1 M KOH (that is later applied as an aqueous electrolyte.

Table S2. Summary of electrochemical performance of CFP electrodes with 1 mol L^{-1} KOH.

**NAME**	**C_{CV}, F g^{-1} 1 mV s^{-1}**	**C_{CV}, F g^{-1} 100 mV s^{-1}**	**RH_$_{CV}$, % ***	**C_{GCPL}, F g^{-1} 0.1 A g^{-1}**	**C_{GCPL}, F g^{-1} 10 A g^{-1}**	**RH_$_{GCPL}$, % ****	**RH_PEIS, % *****	**σ, mS cm^{-1}**	**Time constant, s**
**CFP_195**	187	158	84	197	160	81	77	84	6.2
**CFP_390**	164	115	70	154	106	69	63	157	4.5
**CFP_430**	172	108	63	174	97	56	52	120	16.4
**CFP_750**	183	69	38	186	44	24	24	190	0.6
**CFP_1000**	123	39	31	121	5	4	27	150	0.2
**KCl-T_ele**	175	125	71	173	119	69	52	72	20.9

*RH_$_{CV}$ – rate handling calculated from CV ($C_{100mVs-1}/C_{1mVs-1}$*100%)

**RH_$_{GCPL}$ – rate handling calculated from GCPL ($C_{5Ag-1}/C_{0.1Ag-1}$*100%)

***RH_$_{PEIS}$ – rate handling calculated from PEIS (C_{1Hz}/C_{1mHz}*100%)

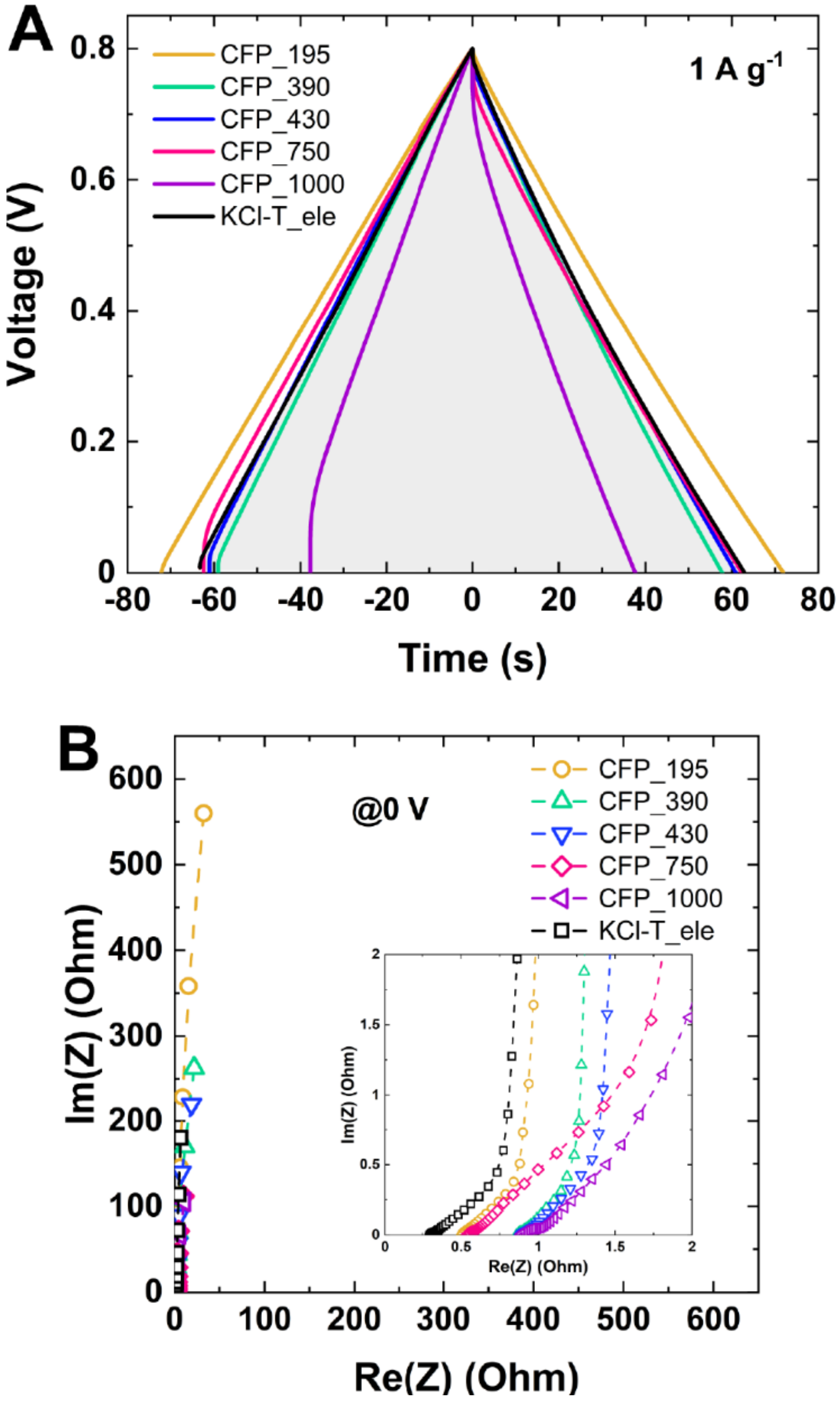

Figure S6. Electrochemical performance of binder free electrodes with 1 mol L^{-1} KOH: A) constant current charge/discharge curve at 1 A g^{-1}; B) Nyquist plot at 0V.

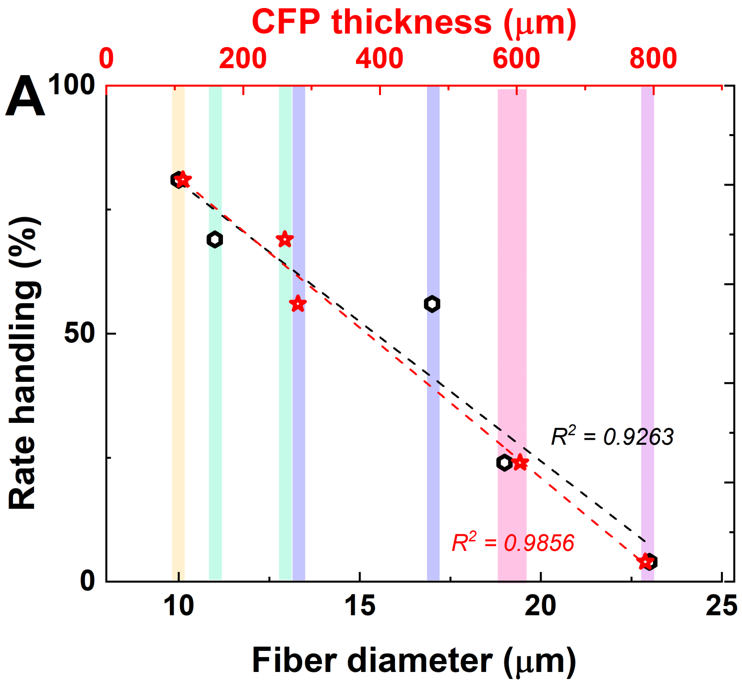

A
CFP thickness (μm)
0
200
400
600
800
100
50
0
Rate handling (%)
R² = 0.9263
R² = 0.9856
10
15
20
25
Fiber diameter (μm)

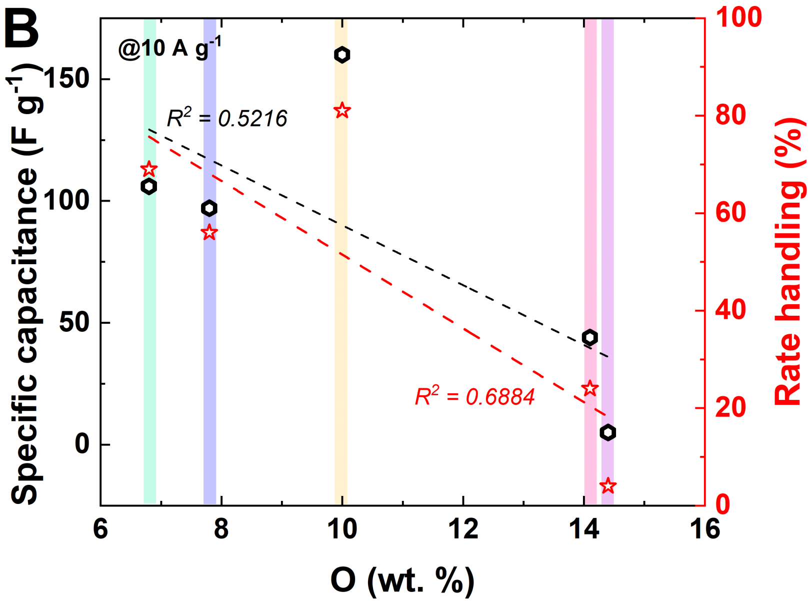

B
@10 A g-1
R² = 0.5216
R² = 0.6884
Specific capacitance (F g-1)
150
100
50
0
Rate handling (%)
100
80
60
40
20
0
6
8
10
12
14
16
O (wt. %)

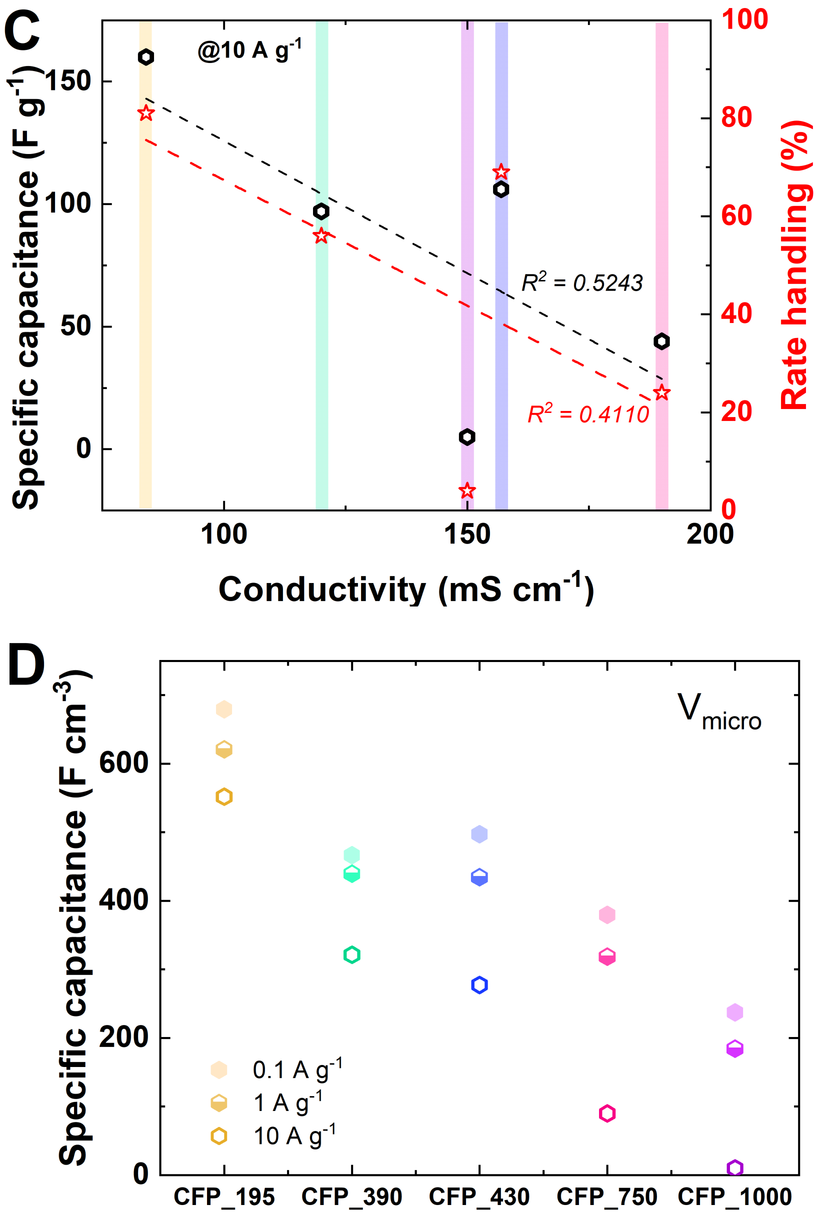

Figure S7. Correlation of physicochemical parameters with electrochemical performance: A) rate handling vs. fiber diameter and CFP thickness; B) specific capacitance and rate handling vs. oxygen content (determined by XPS); C) specific capacitance and rate handling vs. conductivity. Volumetric capacitance of various CFP samples: D) based on V_{micro} at 0.1, 1 and 10 A g^{-1}.

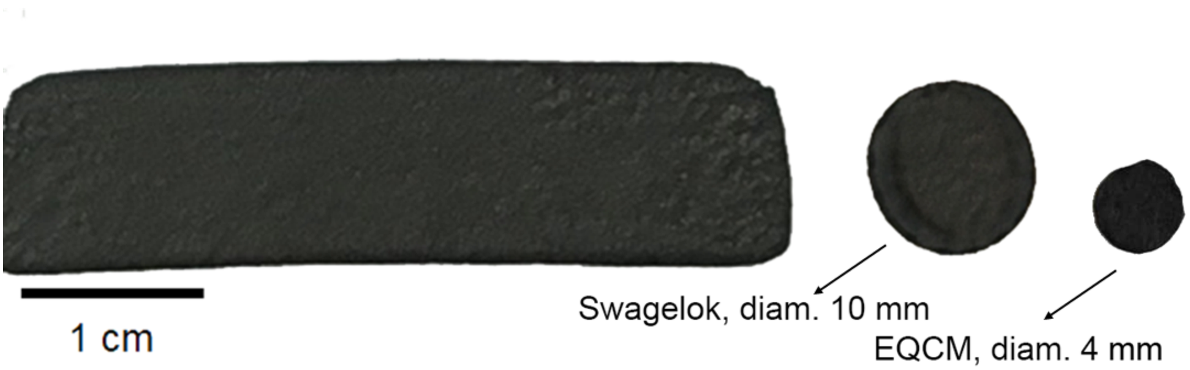

Figure S8. Image of large size binder-free CFP electrode and circular CFP electrodes in scale.

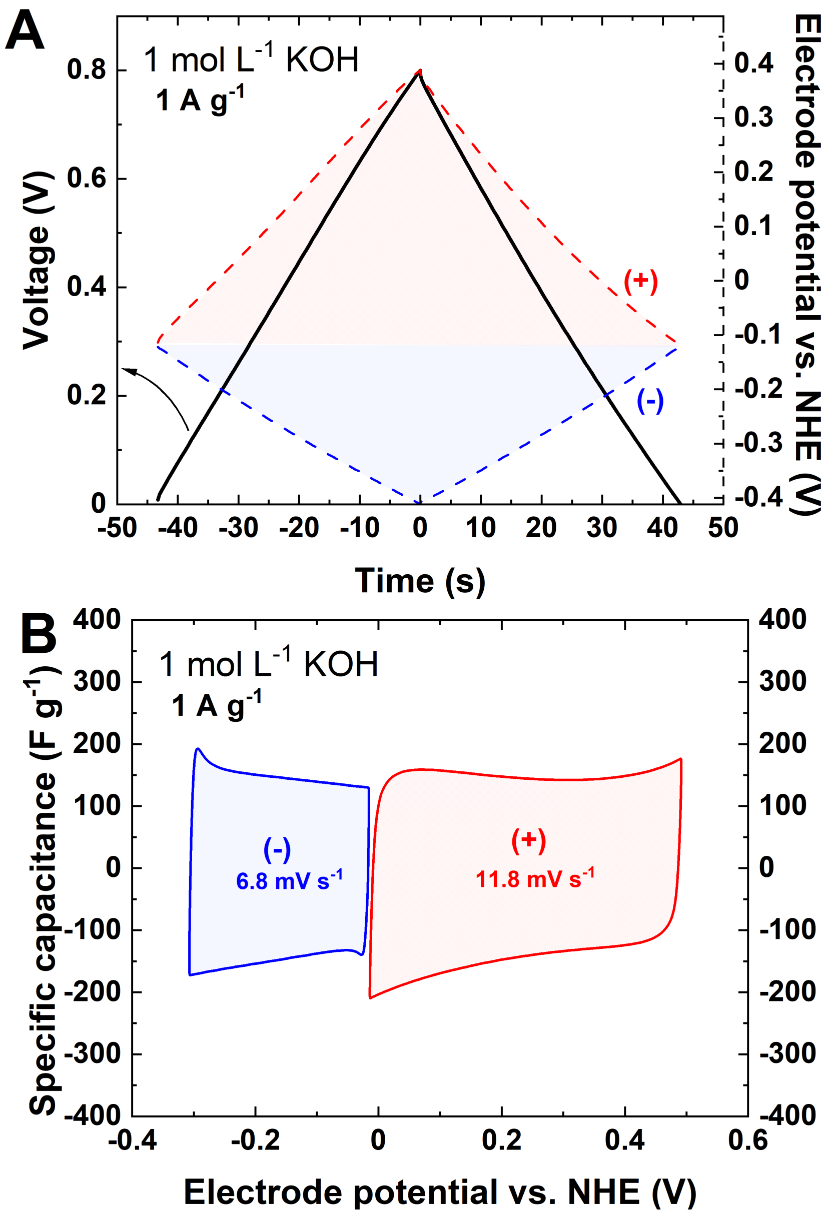

A
1 mol L-1 KOH
1 A g-1
Voltage (V)
Electrode potential vs. NHE (V)
(+)
(-)
Time (s)
B
1 mol L-1 KOH
1 A g-1
Specific capacitance (F g-1)
(-)
6.8 mV s-1
(+)
11.8 mV s-1
Electrode potential vs. NHE (V)

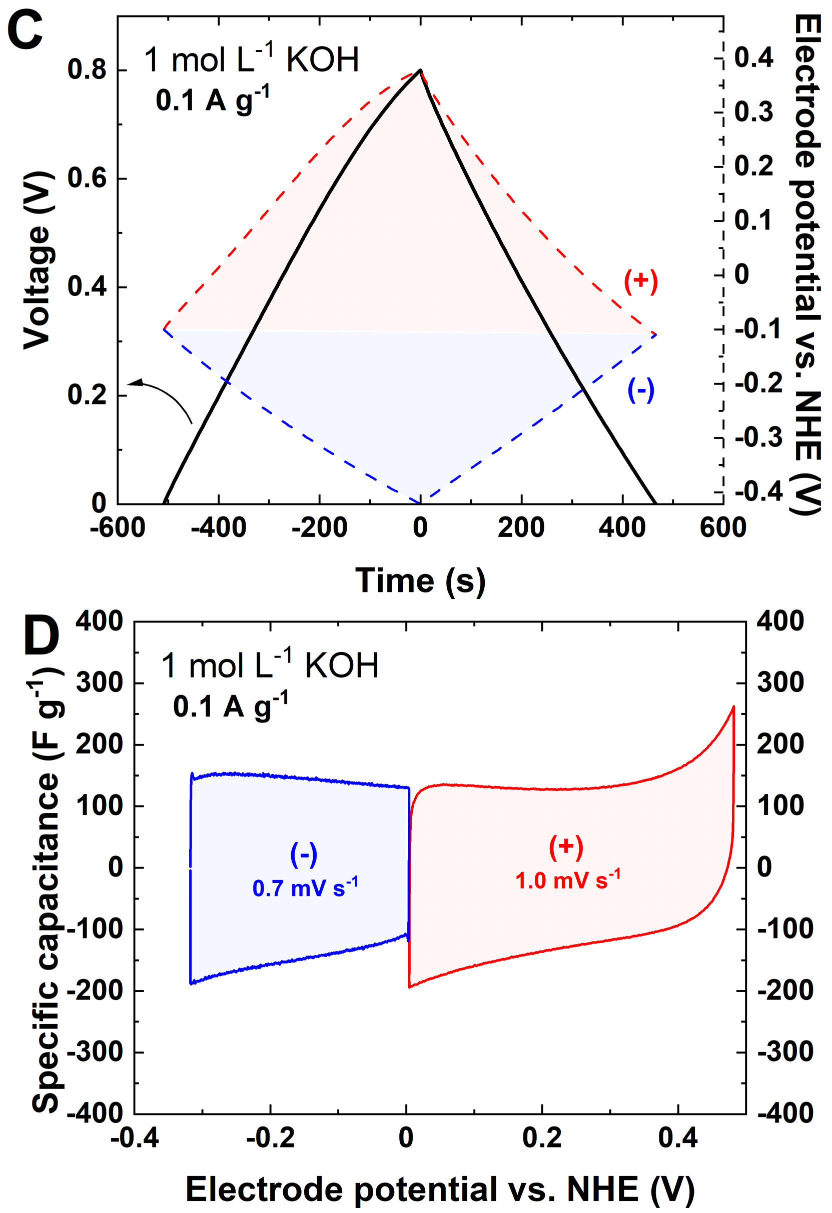

Figure S9. Three-electrode study of electrochemical capacitor with CFP_195 electrodes, operating with 1 mol L^{-1} KOH: A) constant current charge/discharge profile at 0.1 A g^{-1}; B) cyclic voltammograms representing operation of each electrode separately, based on a scan rates cellulated from galvanostatic charge/discharge at 1 A g^{-1}. C) and D) are representative plots for 0.1 A g^{-1}.

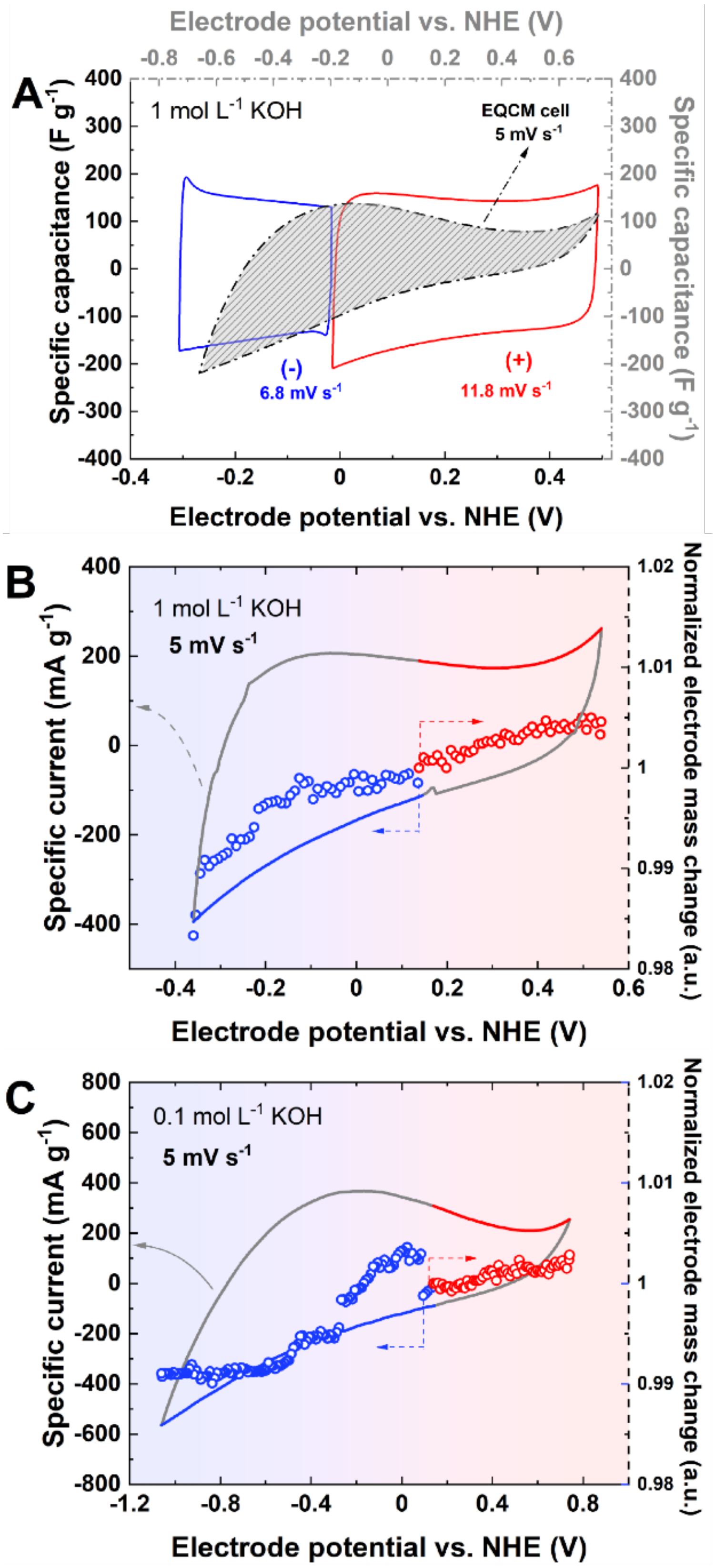

Figure S10. EQCM profiles of CFP_195 electrode: A) cyclic voltammogram recorded in three-electrode set-up and EQCM cell with 1 mol L^{-1} KOH; B) cyclic voltammogram and CFP electrode mass change with 1 mol L^{-1} KOH; C) cyclic voltammogram and CFP electrode mass change with 0.1 mol L^{-1} KOH.

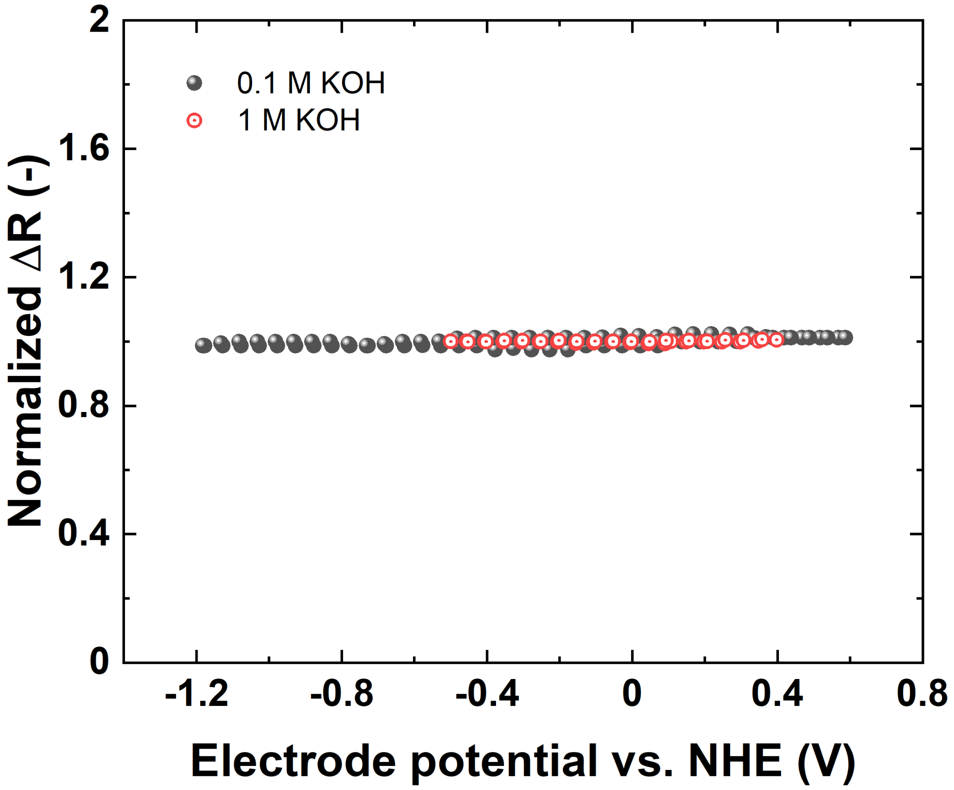

Figure S11. Changes of the resistance during EQCM measurements performed with 1 mol L^{-1} KOH and 0.1 mol L^{-1} KOH electrolytic solutions and CFP_195 electrodes.

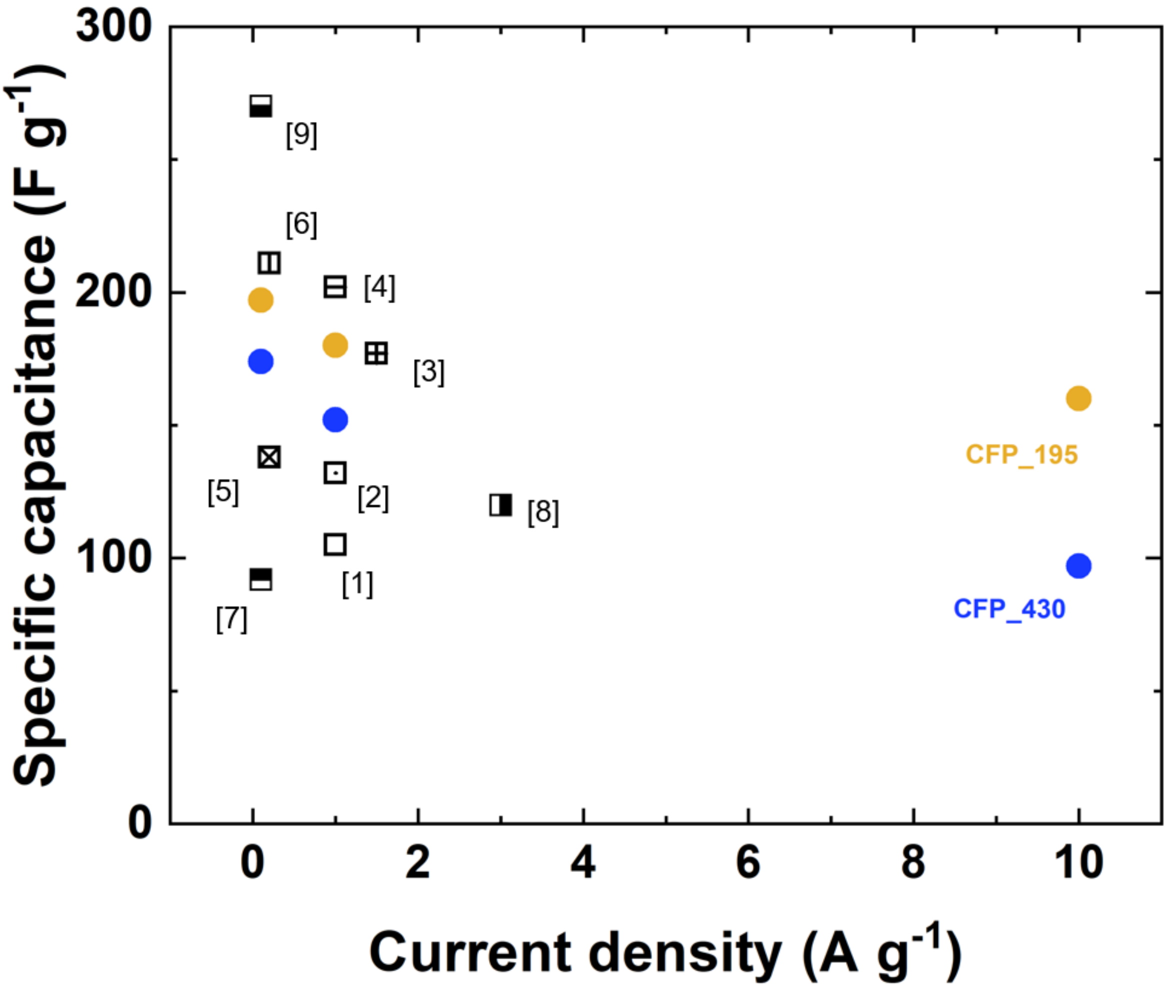

Figure S12. Comparison of CFP_194 and CFP_430 based EC with other reported materials, studied by constant current charge/discharge. Yellow scatter points represent CFP_195, blue scatter points – CFP_430. Black, squared scatters are literature data from [1-9].

1. T. Xiong, W. S. V. Lee, X. Huang and J. M. Xue, 2017, **5**, 12762-12768.
2. U. Kurtan, H. Aydin, B. Buyuk, U. Sahinturk, M. A. Almessiere and A. Baykal, *Journal of energy storage*, 2020, **32**.
3. T. Lavanya and S. Ramaprabhu, *Materials research express*, 2019, **6**, 105005.
4. B. Joshi, S. Park, E. Samuel, H. S. Jo, S. An, M.-W. Kim, M. T. Swihart, J. M. Yun, K. H. Kim and S. S. Yoon, *Journal of electroanalytical chemistry (Lausanne, Switzerland)*, 2018, **810**, 239-247.
5. J. Yan, J.-H. Choi and Y. G. Jeong, *Materials & design*, 2018, **139**, 72-80.
6. H. Liu, W. Song and A. Xing, *RSC advances*, 2019, **9**, 33539-33548.
7. Q. Xie, S. Wu, Y. Zhang and P. Zhao, *Journal of electroanalytical chemistry (Lausanne, Switzerland)*, 2017, **801**, 57-64.
8. K. Song, H. Ni and L.-Z. Fan, *Electrochimica acta*, 2017, **235**, 233-241.
9. W.-L. Song, X. Li and L.-Z. Fan, *Journal*, 2016, **3**, 113-122.
